# PARP1 Inhibitor Combined With Oxaliplatin Efficiently Suppresses Oxaliplatin Resistance in Gastric Cancer-Derived Organoids via Homologous Recombination and the Base Excision Repair Pathway

**DOI:** 10.3389/fcell.2021.719192

**Published:** 2021-08-23

**Authors:** Huafu Li, Chunming Wang, Linxiang Lan, Wenhui Wu, Ian Evans, E. Josue Ruiz, Leping Yan, Zhijun Zhou, Joaquim M. Oliveira, Rui L. Reis, Zhenran Hu, Wei Chen, Axel Behrens, Yulong He, Changhua Zhang

**Affiliations:** ^1^Digestive Diseases Center, The Seventh Affiliated Hospital of Sun Yat-sen University, Shenzhen, China; ^2^Adult Stem Cell Laboratory, The Francis Crick Institute, London, United Kingdom; ^3^The Institute of Cancer Research, London, United Kingdom; ^4^Department of Gastrointestinal Surgery, The First Affiliated Hospital of Sun Yat-sen University, Guangzhou, China; ^5^Center of Scientific Research, The Seventh Affiliated Hospital of Sun Yat-sen University, Shenzhen, China; ^6^Department of Medicine, The University of Oklahoma Health Sciences Center, Oklahoma City, OK, United States; ^7^3B’s Research Group, I3Bs – Research Institute on Biomaterials, Biodegradables and Biomimetics, Headquarters of the European Institute of Excellence on Tissue Engineering and Regenerative Medicine, AvePark, Parque de Ciência e Tecnologia, Zona Industrial da Gandra, University of Minho, Guimarães, Portugal; ^8^ICVS/3B’s – PT Government Associate Laboratory, Guimarães, Portugal

**Keywords:** gastric cancer, L-OHP resistance, homologous recombination, PARP1 inhibitors, organoid

## Abstract

Oxaliplatin (OXA) resistance in the treatment of different types of cancer is an important and complex problem. The culture of tumor organoids derived from gastric cancer can help us to provide a deeper understanding of the underlying mechanisms that lead to OXA resistance. In this study, our purpose was to understand the mechanisms that lead to OXA resistance, and to provide survival benefits to patients with OXA through targeted combination therapies. Using sequence analysis of OXA-resistant and non-OXA-resistant organoids, we found that PARP1 is an important gene that mediates OXA resistance. Through the patients’ follow-up data, it was observed that the expression level of PARP1 was significantly correlated with OXA resistance. This was confirmed by genetic manipulation of PARP1 expression in OXA-resistant organoids used in subcutaneous tumor formation. Results further showed that PARP1 mediated OXA resistance by inhibiting the base excision repair pathway. OXA also inhibited homologous recombination by CDK1 activity and importantly made cancers with normal BRCA1 function sensitive to PARP inhibition. As a result, combination of OXA and Olaparib (PARP-1/2/3 inhibitor), inhibited *in vivo* and *in vitro* OXA resistant organoid growth and viability.

## Introduction

Currently, the standard treatment for gastric cancer is surgical resection. However, the opportunity for surgery is often lost as the majority of cases are diagnosed at an advanced stage (Yuan et al., 2020). Alternative therapies such as radiotherapy and chemotherapy can be considered yet are often ineffective. Available chemotherapy, based on cisplatin and 5-fluorouracil (5-FU) or their combined derivatives, such as oxaliplatin (OXA) and capecitabine, fail in 95% of non-surgical gastric tumors (Wei et al., 2020). In order to advance in the treatment of gastric cancer, there is an urgent need to gain a better understanding of the mechanisms of chemoresistance. This is necessary to provide a more “personalized” treatment to patients and to develop new strategies to overcome chemotherapy resistance.

The use of patient-derived cell lines or xenografts may facilitate the discovery of new therapies because they are closely related to the clinical disease, allowing them to be used to guide chemotherapy selection (Remy et al., 2020). Tumor organoids is an emerging technology that can better mimic primary tumors and provide better tools for *in vitro* research.

Organoids are a three-dimensional (3D) cell culture system derived from primary tissue or stem cells. Compared with most other primary cell cultures, the main advantage of the organoid culture system is that it can maintain the genomic stability of the cell over the long term while maintaining the characteristics of tissue origin (Huch et al., 2015; Liu and Meltzer, 2017). Individual cancer-like organoids can be used to predict therapeutic responses to certain drugs, and the establishment of large gastric cancer organoids biobases in combination with drug screening may help outline new therapeutic strategies for gastric cancer (Lo et al., 2021; Seidlitz et al., 2021). When gastric cancer organoids are exposed to commonly used chemotherapy agents, there are varying degrees of response, comparable to the clinical response of the patient (Wang et al., 2014; Yan et al., 2018). Thus we can use patient derived organoids to investigate the mechanisms of OXA resistance.

In this study, we aim to analyze the clinical samples of patients and conduct drug sensitivity experiments with gastric cancer organoids. Clinical specimens of four gastric cancer patients were obtained and organoid cultures established, two from patients who had responded well to OXA treatment and two whose tumors were OXA-resistant.

*In vivo* and *in vitro* studies using the organoids mirrored the clinical data in terms of OXA sensitivity. Sequencing data suggested PARP1 as a key gene involved in mechanisms of OXA resistance and this was confirmed using a range of *in vitro* and *in vivo* approaches. Importantly, we demonstrated that combining OXA with a PARP1 inhibitor is able overcome the OXA resistance and points the way to a potential new therapeutic modality for the treatment of GC.

## Results

### PARP1 Is an Important Core Gene Leading to OXA Resistance

**FIGURE 1:**
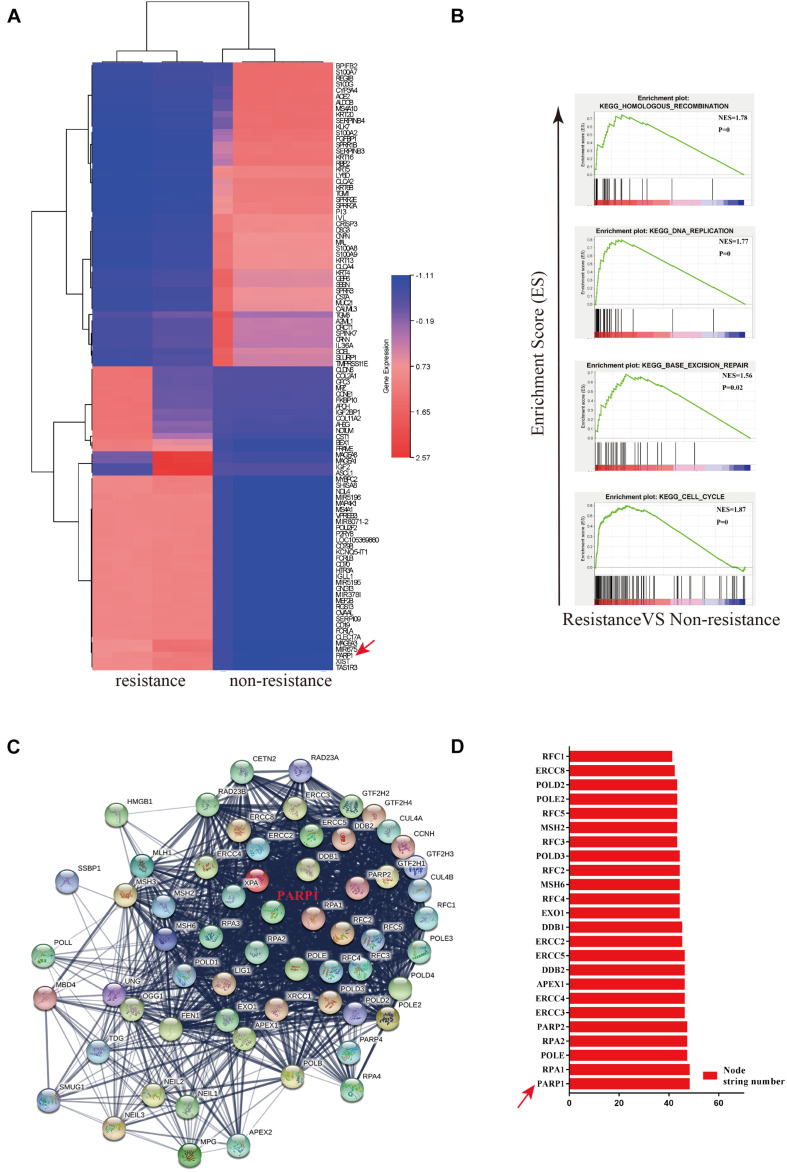
PARP1 is the central gene of Oxaliplatin resistance in gastric cancer. **(A)** Heatmap of mRNA differential expression of sGC1 and sGC2 tumors against rGC1 and rGC2 tumors. The abscissa represents the gene name. Red represents High and blue represents Low. **(B)** Analysis of enrichment of mRNA differential expression of sGC1 and sGC2 tumors against rGC1 and rGC2 tumors. **(C)** STRING database protein interaction network diagram of mRNA differential expression in sGC1 and sGC2 tumors compared to rGC1 and rGC2 tumors. Edges represent protein-protein associations. Cambridge blue, curated databases; Violet, experimentally determined; Green, gene neighborhood; Red, gene fusions; Blue, gene co-occurrence; Reseda, text mining; Black, co-expression; Lilac, protein homology. **(D)** Comparison of NODE string number of two gene sets in core genes of **(C)**. All experiments were repeated three times.

In order to investigate the mechanisms behind the chemotherapuetic resistance, we used four patient-derived organiods (sGC1, sGC2, rGC1, and rGC2), rGC1 and rGC2 were derived from patients whose GC recurred after postoperative chemotherapy, while sGC1 and sGC2 were from patients without recurrence after postoperative chemotherapy. In a viability assay, rGC1 (IC50 = 19.95 μm.L^–1^) and rGC2 (IC50 = 63.09 μm.L^–1^) were found to be more resistant to OXA than sGC1 (IC50 = 0.93 μm.L^–1^) and sGC1 (IC50 = 3.03 μm.L^–1^) (Supplementary Figure 1A). In order to explore the regulation of core genes that may mediate OXA-resistance, we performed mRNA sequencing on the patient derived organoids. The data was anlysed by PPi network construction. Figure 1A shows their differentially expressed genes (DEGs) and fold change (FC). Compared with non-drug-resistant patients, the main enrichment pathways for drug-resistant patients include homologous recombination (HR), DNA replication, base excision repair (BER), and cell cycle regulation (Figure 1B). Finally, we searched for the core genes using String and found that PARP1 was a candidate gene affecting drug resistance (Figures 1C,D).

### PARP1 Is Upregulated in Gastric Cancer OXA Resistance Organoid

**FIGURE 2:**
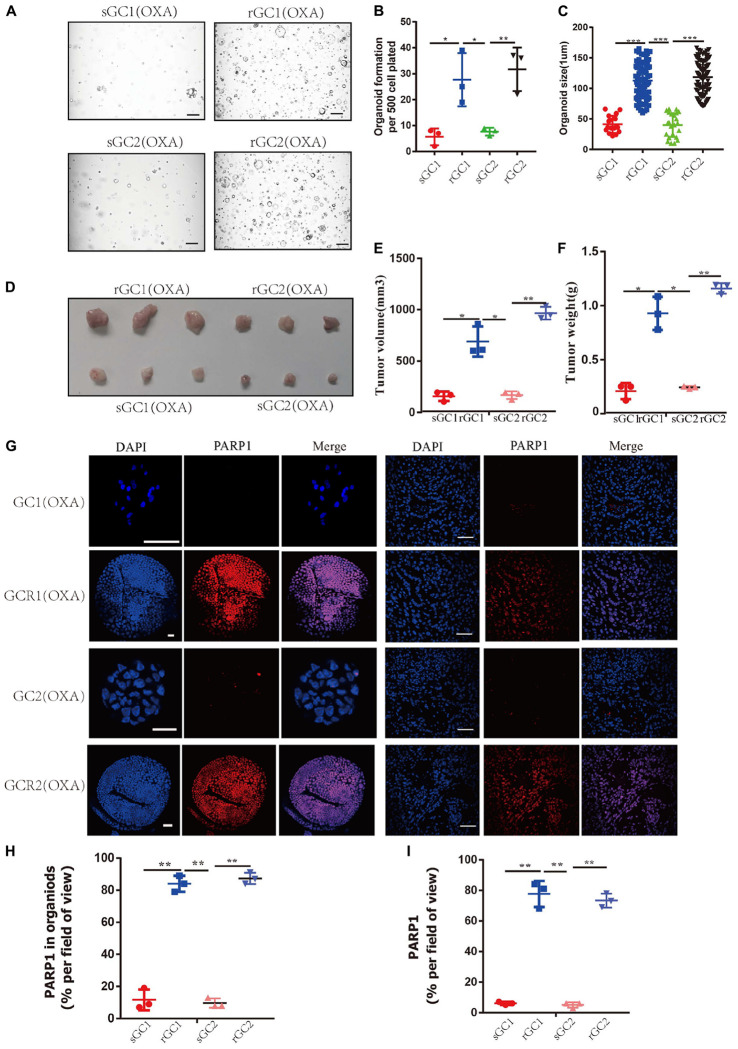
PARP1 is upregulated in oxaliplatin resistance gastric cancer. **(A**–**C)** sGC1, sGC2, rGC1, and rGC2 organoids were treated with Oxaliplatin before imaging **(A)**, number of organoids **(B)**, and size of organoids **(C)**. **(D)** Representative images of tumorigenesis in BALB/C NUDE mice treated with Oxaliplatin. The ruler represents 1 cm. **(E)** Tumour growth curves of organoids in PDOX BALB/C Nude mice. The curve shows the average tumor volume. Error bars represent mean ± standard deviation. **(F)** Mass of tumors of PDOX in BALB/C NUDE mice. The curve shows the average tumor mass. **(G)** Representative images of PARP1 levels stained by immunofluorescence in organoid and tumors. The scale bar represents 20 μm for tumour images, 200 μm for organoid images. **(H)** Proportion of PARP1 + cells in organoid **(G,I)**, proportion of PARP1 + cells in tumor **(G)**. The Student’s *t* test was used for statistical analysis. Error bars indicate mean ± standard deviation. *< 0.05, **< 0.01, and ***< 0.001. All experiments were repeated three times.

Through our experiments on OXA resistance of rGC1, rGC2, sGC1 and sGC2 *in vitro* and *in vivo*, it was found that the tolerance of rGC1 and rGC2 to OXA was significantly higher than that of sGC1 and sGC2 (Supplementary Figure 1D and Figures 2A–F). Moreover, it was found that the expression level of PARP1 in rGC1 and rGC2 of OXA-resistant organoid was significantly higher than that in sGC1 and sGC2 of OXA-sensitive organoid (*P* < 0.05) (Figures 2G–I).

### PARP1 Plays an Important Role in Maintaining OXA Resistance

**FIGURE 3:**
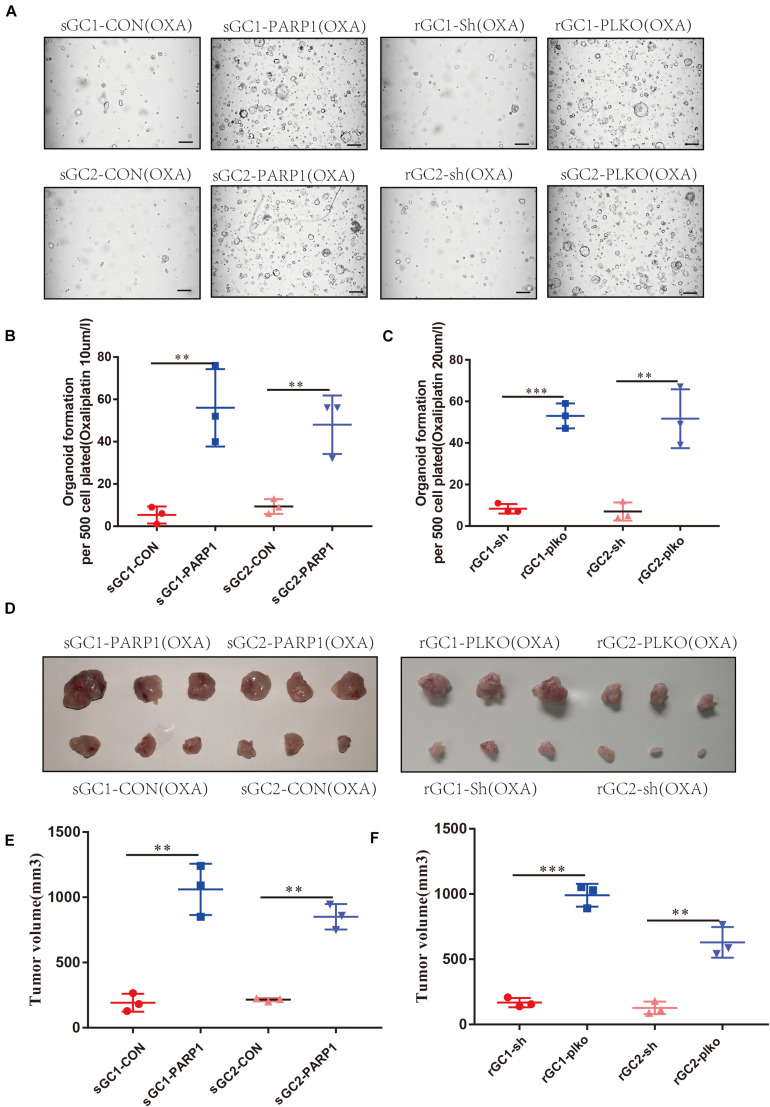
PARP1 is required for Oxaliplatin resistance development. **(A–C)** sGC1, sGC2 and sGC1 PARP1 overexpression, sGC2 overexpression, rGC1, rGC2 and rGC1 PARP1 knock-down, and rGC2 knock-down organoids were treated with Oxaliplatin before imaging **(A)** number of organoids **(B,C)**. **(D)** Representative images of tumorigenesis in BALB/C NUDE mice with Oxaliplatin. The ruler represents 1 cm. **(E)** Tumor growth curves of PDOX BALB/C NUDE mice of sGC1, sGC2 and sGC1 PARP1 overexpression, and sGC2 overexpression. The curve shows the average tumor volume. Error bars represent mean ± standard deviation. **(F)** Tumor growth curves of PDOX BALB/C NUDE mice of rGC1, rGC2 and rGC1 PARP1 knock-down, and rGC2 knock-down. The curve shows the average tumor volume. Error bars represent mean ± standard deviation. Three mice carried xenografts and one xenograft per mouse. All experiments were repeated three times. *< 0.05, **< 0.01, and ***< 0.001.

To confirm the role of PARP1 in OXA resistance, we overexpressed PARP1 in the OXA-sensitive organoids and found that this increased their tolerance to OXA. Conversely, PARP1 knockdown in OXA-resistant organoid, showed decreased tolerance to OXA (*P* < 0.05) (Figures 3A–C and Supplementary Figure 3A). Similar results were seen *in vivo* when PARP1 was overexpressed in the OXA-sensitive organoid before subcutaneous implantation, and it was found that the tolerance of the resulting tumors to OXA was significantly increased. Moreover, PARP1 knockdown was performed on the OXA-resistant rGC1 and rGC2 organoids and it was found that the tolerance of tumors to OXA *in vivo* was reduced (*P* < 0.05) (Figures 3D–F). These results indicate that PARP1 plays a pivotal role in OXA resistance *in vitro* and *in vivo.*

### PARP1 Inhibition by Olaparib Sensitizes Gastric Cancer to OXA

**FIGURE 4:**
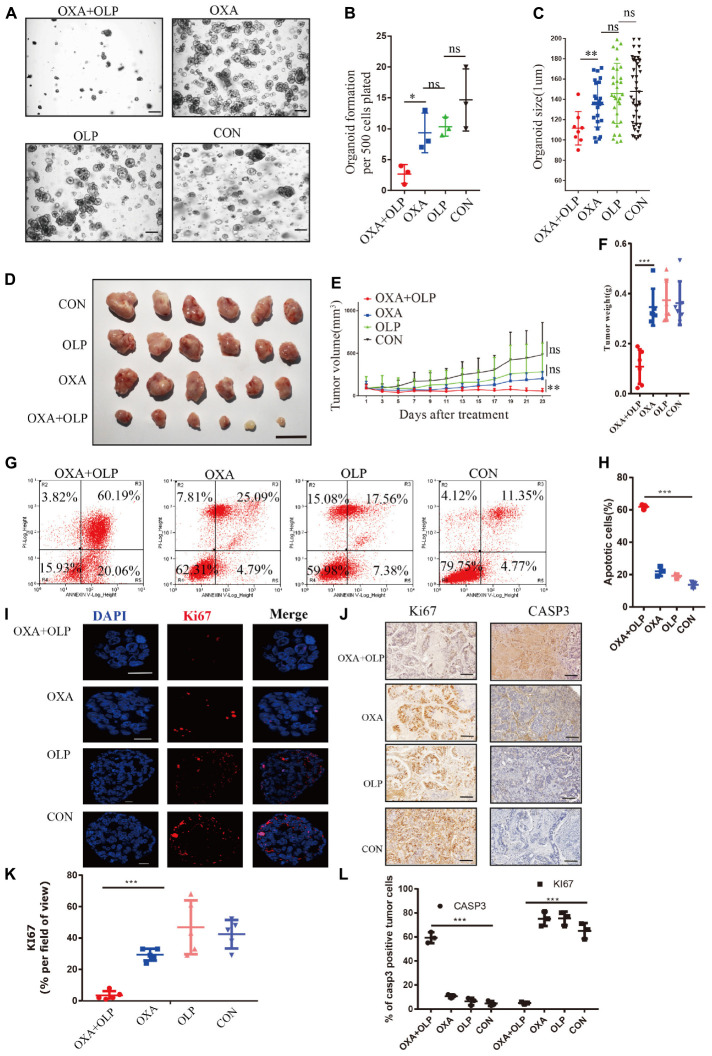
PARP1 inhibition by Olaparib sensitizes gastric cancer to Oxaliplatin. **(A–C)** rGC1 organoids were treated with Olaparib + Oxaliplatin, Oxaliplatin, and Olaparib, respectively, before imaging **(A)**, number of organoids **(B)**, and size of organoids **(C)**. The scale represents 500 μm. Different drug concentrations were used to act on cells and before cell viability tests **(A,E)**. Drug action time was 36 h. **(D)** Representative images of tumorigenesis in BALB/C NUDE mice with Olaparib + Oxaliplatin, Oxaliplatin, Olaparib and a blank control group of rGC1. The ruler represents 1 cm. **(E)** Tumor growth curves of PDOX BALB/C NUDE mice of rGC1 organiod. The curve shows the average tumor volume. Error bars represent mean ± standard deviation. **(F)** Mass of tumors of PDOX in BALB/C NUDE mice of rGC1 organiod. The curve shows the average tumor mass. **(G)** The effect of different medication groups on the apoptosis of rGC1 organoids. **(H)** Comparison of the proportion of apoptosis in different groups. **(G,I)** Representative images of KI67 stained by IF staining of rGC1 organoids treated with olaparib + oxaliplatin, oxaliplatin, Olaparib, and a blank control group. The red stains indicate KI67 positive. The scale represents 2 um. **(J)** Representative images of KI67 and Caspase3 stained by IHC staining after tumorigenesis of BALB/C NUDE mice treated with Olaparib + Oxaliplatin, Oxaliplatin, Olaparib, and a blank control group of rGC1. The brown stains indicate KI67 and Caspase3 positive. The scale represents 200 um. **(K)** Statistical analysis of KI67 + cells. **(I,L)** Comparison of the percentage of positive cells stained with KI67 and Caspase3. Student’s *t* test was used for statistical analysis. Error bars indicate mean ± standard deviation. OXA, Oxaliplatin. OLP, Olaparib. CON, control group. *< 0.05, **< 0.01, and ***< 0.001. All experiments had repeated three times. PDOX, patient-derived organiod culture xenograft. ns, no significant.

Since PARP1 appears be an important gene for OXA resistance we wanted to determine whether a PARP1 inhibitor combined with OXA can effectively inhibit OXA resistance. Using both the PARP1 inhibitor, Olaparib, and OXA in combination effectively inhibited the viability, size, cell count, and proliferation of the organoids derived from the OXA resistance gastric cancers (rGC1 and rGC2) (*P* < 0.05) (Figures 4A–C). This drug combination also significantly inhibited the activity and proliferation of a range of OXA resistance gastric cancer cell lines (Supplementary Figures 2A,B). BALB/C NUDE mice *in vivo* tumorigenesis experiments also confirmed that these drugs when used in combination could effectively inhibit tumor growth when compared with their use individually (*P* < 0.05) (Figures 4D–F), and can induce cell apoptosis and affect proliferation of tumor cells (*P* < 0.05) (Figures 4G–L). By comparing Olaparib + OXA versus OXA alone, it was found that the Olaparib + OXA group was mainly enriched in oxidative phosphorylation and PPAR signaling pathway (Supplementary Figures 2C,D). These two pathways are primarily important enrichment pathways for tumor apoptosis after chemotherapy-induced DNA damage (Yang and Frucht, 2001; Yadav et al., 2015). The main pathways enriched in the OXA group were JAK-STAT, MAPK, NOTCH, and WNT signaling pathways (Supplementary Figures 2E–H). In fact, these pathways are not only related to drug resistance in tumors, but also closely related to proliferation.

### Combined Oxaliplatin With Olaparib Inhibits BER and HR Repair Pathways via Blocking Both CDK1-BRCA1 and PARP1-Related Activities

**FIGURE 5:**
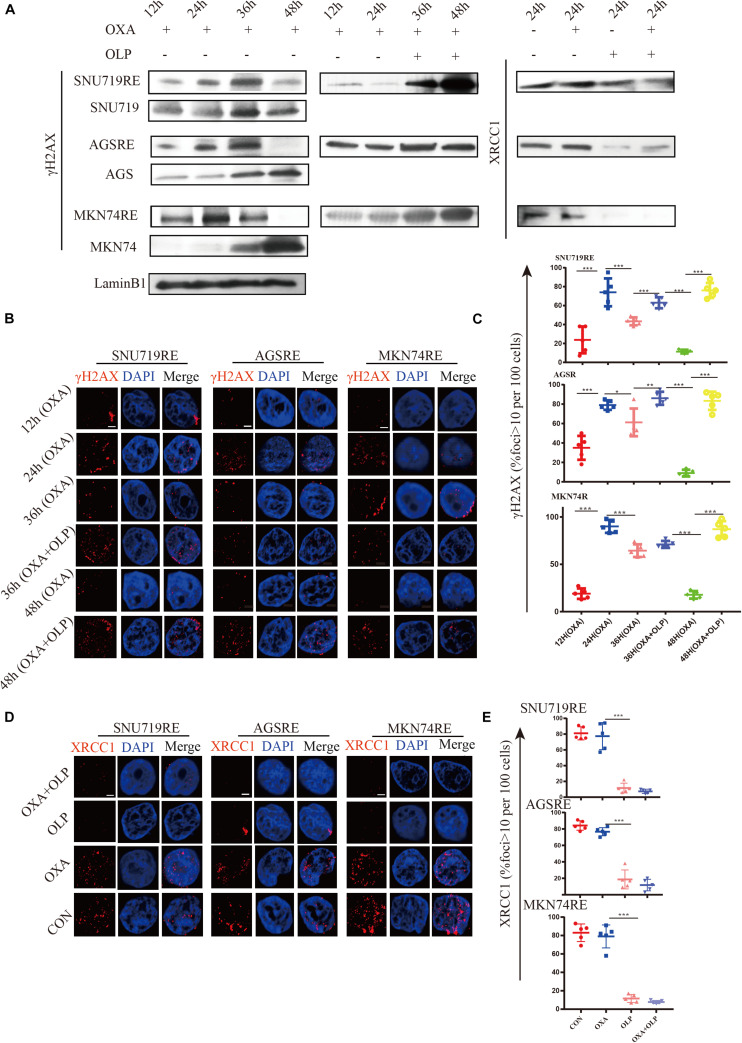
The inhibition of PARP1 can significantly enhance the DNA damage and inhibit BER of Oxaliplatin-resistant GC. **(A)** Comparison of γH2AX expression in Olaparib + Oxaliplatin, Oxaliplatin, Olaparib, and the blank control group and the MKN74, SNU719, AGS resistant strains, and their corresponding wild-type cell lines at different times. Comparison of XRCC1 expression in Olaparib + Oxaliplatin, Oxaliplatin, Olaparib, and the blank control group in MKN74, SNU719, and AGS Oxaliplatin resistance strains. **(B)** Immunofluorescent comparison of γH2AX expression in Olaparib + Oxaliplatin, Oxaliplatin, Olaparib, and the blank control group and the MKN74, SNU719, AGS resistant strains, and their corresponding wild-type cell lines at different times. **(C)** Ratio of γH2AX + cells in MKN74, SNU719, AGS Oxaliplatin resistance strains, and their corresponding wild-type cell line **(B,D)** immunofluorescent comparison of XRCC1 expression in Olaparib + Oxaliplatin, Oxaliplatin, Olaparib, and the blank control group in MKN74, SNU719, and AGS Oxaliplatin resistance strains. The scale represents 2 um. **(E)** Ratio of XRCC1 cells in MKN74, SNU719, AGS Oxaliplatin resistance strain and their respective wild-type cell lines **(D)**, respectively. The Student’s *t* test was used for statistical analysis. Error bars indicate mean ± standard deviation. OXA, Oxaliplatin. OLP, Olaparib. CON, control group. AGSR, AGS Oxaliplatin resistance. SNU719R, SNU719 Oxaliplatin resistance. MKN74R, MKN74 Oxaliplatin resistance. *< 0.05, **< 0.01, and ***< 0.001. All experiments were repeated three times.

Through the study above, the increase in PARP1 expression was found to be an important mediating factor for OXA resistance. We then investigated the mechanims of PARP1 mediated OXA resistance. PARP1 is usually used to repair single base breaks in DNA, which are a type of commonly occurring DNA damage, and not normally harmful to cells. However, when these broken bases are transcribed or replicated, they will destroy and cause damage to the new DNA copies. The activation of PARP1 can promote DNA base excision repair (BER) and inhibit the binding of transcription factors to single-stranded DNA, thus inhibiting the transcription of damaged DNA (Slyskova et al., 2018). PARP1 is highly likely to mediate OXA resistance through its regulation of DNA repair mechanisms. First, the effect of OXA on DNA damage (increase of γH2AX) in OXA resistant cells and sensitive cells was studied and the results showed that the resistant cells were able to effectively repair DNA (decrease of γH2AX) after the damage by OXA (Figure 5A). However, when PARP1 was inhibited in these cells, DNA repair was significantly impaired (*P* < 0.05) (Figures 5B,C). The role of BER in cancer drug resistance had been proposed by many studies (Horton et al., 1995; Faivre et al., 2003; Preston et al., 2009; Yang et al., 2010; Sawant et al., 2017), and PARP1 plays an important role in the BER pathway (Ronson et al., 2018). To this end, the effect of PARP1 inhibiton combined with OXA on the BER pathway marker, XRCC1 was studied, and Olaparib + OXA was found to significantly inhibit the BER pathway when compared to OXA alone (Figures 5A,D,E). However, the transcription levels of XRCC1 in OXA resistance patients and non-resistant patients, and XRCC1 of OXA resistance and non-resistant cell lines did not change significantly (Supplementary Figures 3B,C).

The role of PARP1 is to bind to DNA damage sites (mostly single-stranded DNA breaks) and catalyze the synthesis of poly ADP ribose chains on protein substrates (Mateo et al., 2019). In order to study the core target of PARP1 interaction, weighted gene co-expression network analysis (WGCNA) was used to find the core gene that interacted with PARP1. The data indicated that CDK1 (Cyclin-dependent kinase) played a key role in the high expression of PARP1 (Supplementary Figure 4 and Supplementary Table 1). CDK 1 is a core component of the cell cycle mechanism, forming a complex with cyclin A and B to promote the progression of S phase, G2 phase and M phase. Recently, CDK1 and its other family members have been shown to be involved in the DNA damage response pathway (Myers et al., 2007). Studies have found that CDK1 can inhibit homologous recombination by inhibiting the phosphorylation of BRCA1 (Johnson et al., 2009, 2011). Thus, we next determined whether OXA can directly act on BRCA1 or CDK1 to inhibit BRCA1 and cause homologous recombination failure.

**FIGURE 6:**
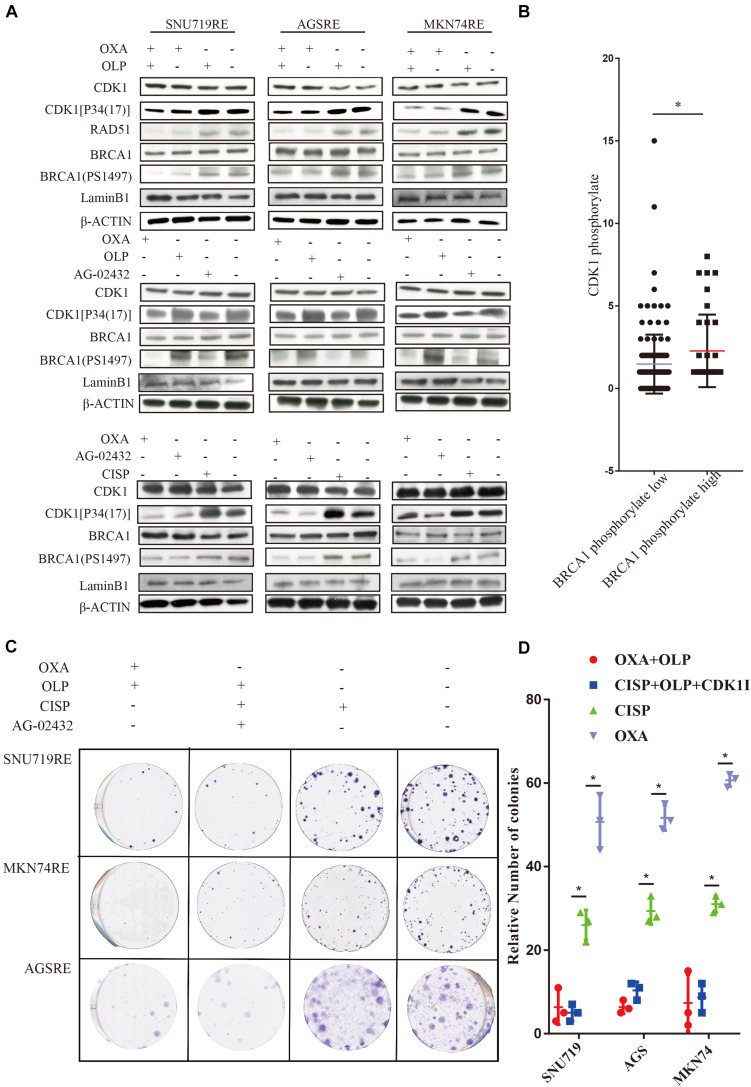
Treatment of Oxaliplatin inhibits HR repair pathways via blocking CDK1-BRCA1 activities in Oxaliplatin resistance cell line. **(A)** Verification by WB on the effects of Olaparib + Oxaliplatin, Oxaliplatin, Olaparib, AG-02432 and cisplatin on CDK1 expression and its phosphorylation, BRCA1 expression and its phosphorylation, RAD51 expression in SNU719, MKN74, and AGS Oxaliplatin resistance strains. Drug action time was 36 h. **(B)** Histochemical results of protein phosphorylation in gastric cancer patients. **(C,D)** The effects of Olaparib + Oxaliplatin and cisplatin combined with CDK1 inhibitor Olaparib on colony formation of overexpressed PARP1 and normally expressed PARP1 cell lines in SNU719, MKN74, and AGS Oxaliplatin resistance strains. Colonies were stained with crystal violet. The Student’s *t* test was used for statistical analysis. Error bars indicate mean ± standard deviation. OXA, Oxaliplatin. OLP, Olaparib. CON, control group. CISP, cisplatin. PCDK1, CDK1 phosphorylation antibody. PBRCA1, BRCA1 phosphorylation antibody. AGSR, AGS Oxaliplatin resistance. SNU719R, SNU719 Oxaliplatin resistance. MKN74R, MKN74 Oxaliplatin resistance. ^∗^< 0.05. All experiments were repeated three times.

**FIGURE 7:**
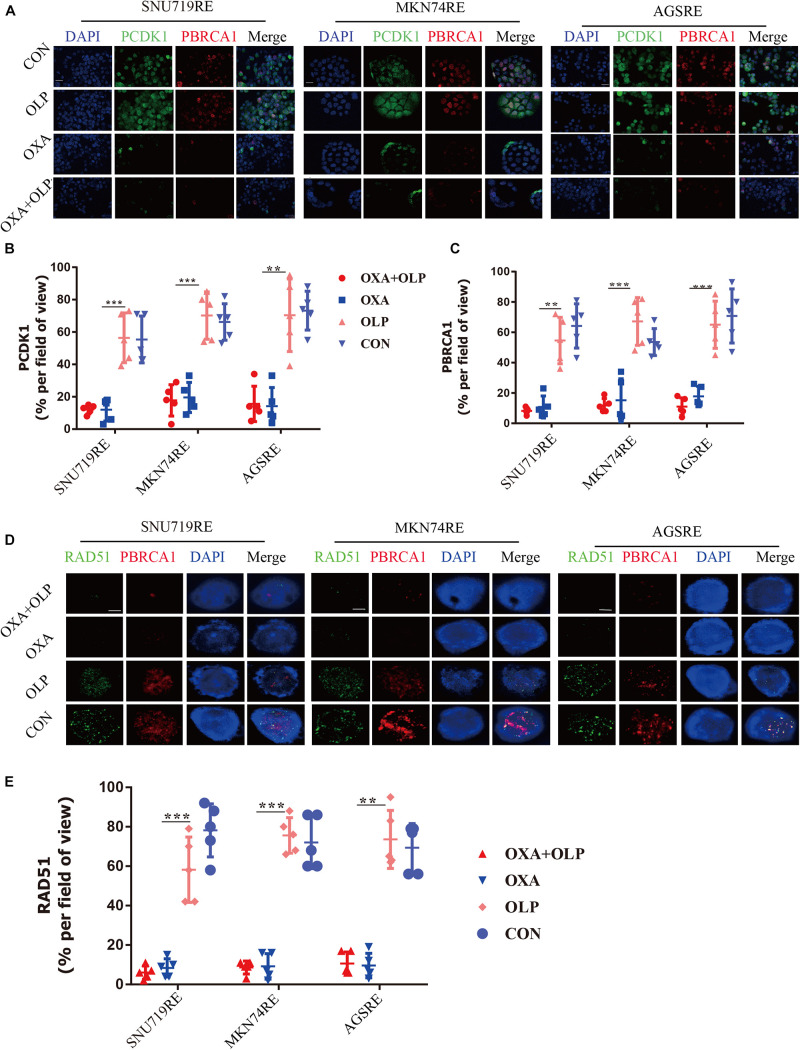
Oxaliplatin inhibits HR repair pathways via blocking both CDK1-BRCA1. **(A)** Representative images of immunofluorescent CDK1 phosphorylation and BRCA1 phosphorylation staining of Olaparib + exaliplatin, Oxaliplatin, Olaparib, and blank control group in MKN74, SNU719, AGS Oxaliplatin resistance cell lines. The scale represents 20 um. Drug action time was 36 h. **(B,C)** Proportion of CDK1 phosphorylation and BRCA1 phosphorylation positive cells **(A)**, respectively. **(D)** Representative images of immunofluorescent phosporylation staining comparisons of Olaparib + Oxaliplatin, Oxaliplatin, Olaparib and blank control group and immunofluorescent phosphorylation staining of RAD51 and BRCA1 of MKN74, SNU719, AGS resistant strains RAD51, and BRCA1 at different time. The scale represents 2 um. **(E)** Statistical analysis of RAD51 + cells in **(D)**. The Student’s *t* test was used for statistical analysis. Error bars indicate mean ± standard deviation. OXA, Oxaliplatin. OLP, Olaparib. CON, control group. PCDK1, CDK1 phosphorylation antibody. PBRCA1, BRCA1 phosphorylation antibody. AGSRE, AGS Oxaliplatin resistance. SNU719RE, SNU719 Oxaliplatin resistance. MKN74RE, MKN74 Oxaliplatin resistance. *< 0.05, **< 0.01, and ***< 0.001. All experiments were repeated three times.

**FIGURE 8:**
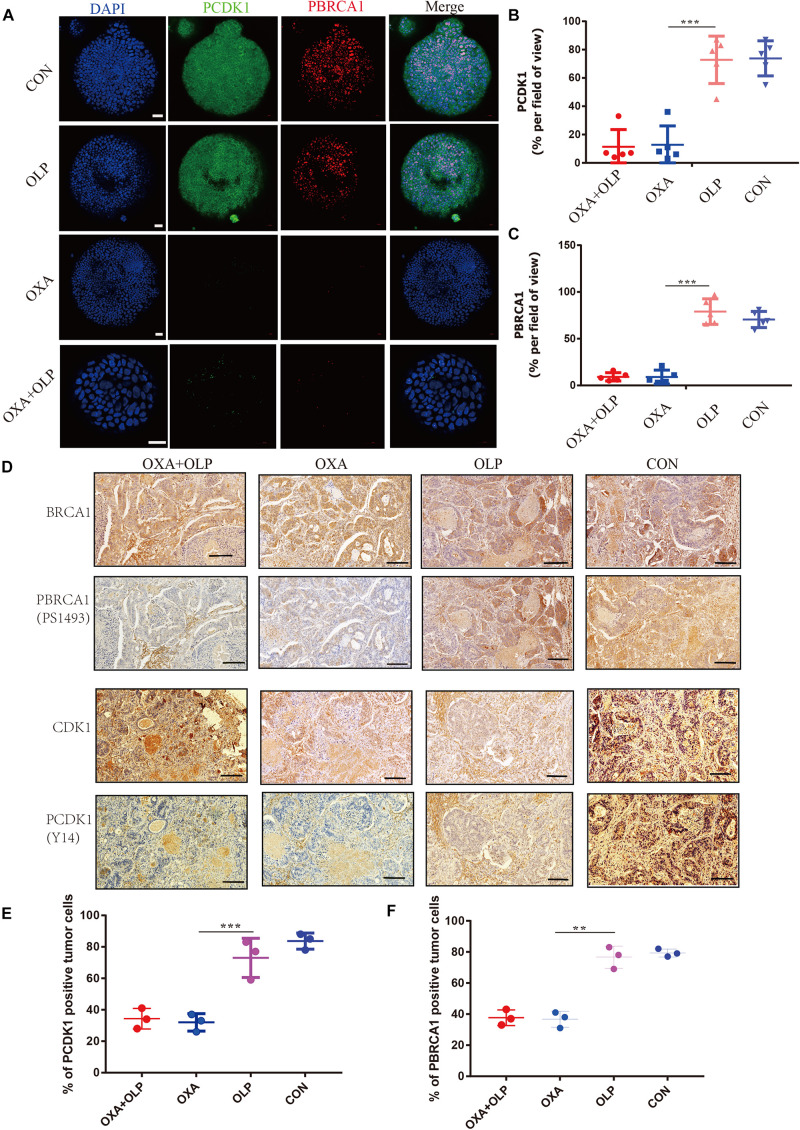
Treatment of Oxaliplatin inhibits HR repair pathways via blocking CDK1-BRCA1 activities in Oxaliplatin resistance gastric cancer Organoid and PDOX. **(A)** Representative images of immunofluorescent staining comparison of CDK1 phosphorylation and BRCA1 phosphorylation in öxaliplatin resistance gastric cancer organoids under the effects of Olaparib + Oxaliplatin, Oxaliplatin, Olaparib, and the blank control group. The scale represents 20 μm. Drug action time was 36 h. **(B,C)** Proportion of CDK1 phosphorylation and BRCA1 phosphorylation positive cells **(A)**, respectively. **(D)** Representative images of comparison of IHC staining of CDK1 and its phosphorylation and BRCA1 and its phosphorylation in BALB/C NUDE mice after tumorigenesis under the effects of Olaparib + Oxaliplatin, Oxaliplatin, Olaparib, and blank control group. The scale represents 200 um. **(E,F)** Proportion of CDK1 phosphorylation and BRCA1 phosphorylation positive cells in **(D)**, respectively. The Student’s *t* test was used for statistical analysis. Error bars indicate mean ± standard deviation. OXA, Oxaliplatin. OLP, Olaparib. CON, control group. CISP, cisplatin. PCDK1, CDK1 phosphorylation antibody. PBRCA1, BRCA1 phosphorylation antibody. PDOX, patient-derived organotipic culture xenograft. **< 0.01, and ***< 0.001. All experiments were repeated three times.

To do this, we investigated the interaction of OXA with BRCA1 and CDK1. First, the Olaparib + OXA drug combination was compared with single drug OXA. OXA was seen to significantly inhibit the phosphorylation of BRCA1 and CDK1 (Figures 6A,B), but Olaparib had no significant effect (Figures 6A, 7A–C, 8A–F). In addition to affecting the functions of BRCA1 and CDK1, OXA also decreased the expression level of RAD51 (*P* < 0.05) (Figures 6A, Figure 7D,E). RAD51 is an important marker in homologous recombination. OXA may be able to inhibit homologous recombination by affecting the function of BRCA1, which in turn leads to a decrease in RAD51 and ultimately aggravating DNA damage (such as increased expression of H2AX). But whether OXA indirectly inhibited BRCA1 function by inhibiting CDK1 or directly inhibiting BRCA1 function remains unclear. So their relationship was compared by inhibiting CDK1. Figure 6A showed that CDK1 inhibitors significantly decreased the phosphorylation of BRCA1, and the effect was similar to that of OXA. In order to examine whether OXA can bypass CDK1 and directly inhibit BRCA1, the functional effects of cisplatin, which is also a platinum-based drug, was used on CDK1 and BRCA1 and compared to that of OXA. It was found that cisplatin did not inhibit the functions of CDK1 and BRCA1 (Figure 6A). Moreover, it was shown through proliferation and colony formation assay that the effect of cisplatin combined with PARP1 and CDK1 inhibitors was not significantly different from the effect of OXA combined with PARP1 inhibition (Figures 6C,D). Thus CDK1 plays an important role in killing tumor cells in platinum-based chemotherapy. In fact, although the principle of action of Cisplatin and OXA is basically the same, cisplatin is less effective than that of OXA (Liu et al., 2019) and CDK1 may be the main reason for this difference. Since OXA, in combination with olaparib, works by inhibiting both BER and HR. We further verified our results by comparing their effects on BER and HR markers as well as DNA damage markers through combination of drugs (Supplementary Figures 5A–D). It was found that that OXA inhibited HR and olaparib inhibited BER, which together leads to the aggravation of DNA damage in cells.

### PARP1 Expression Predicts the Relapse of Human Gastric Cancer After Surgery

**FIGURE 9:**
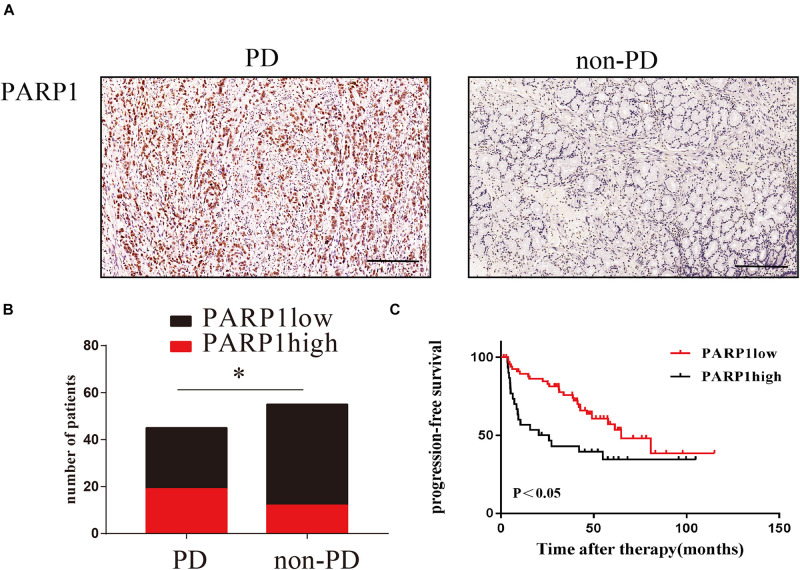
PARP1 expression predicts the relapse of human gastric cancer after surgery. **(A)** Representative images of IHC stained PARP1 in PD tumors (left) and non-PD tumors (right). The brown nucleus is positive for PARP1. The scale represents 200 um. **(B)** Comparison of the number of patients with PARP1 positive and negative staining. **(C)** Comparison of PARP1 expression and tumor recurrence time after chemotherapy. Kaplan–Meier survival plot was used. *< 0.05. PD, progressed disease.

In order to clinically verify the importance of PARP1 in the recurrence of gastric cancer after curative surgery and adjuvant chemotherapy, we enrolled gastric cancer patients undergoing adjuvant chemotherapy in Sun Yat-sen University’s Gastric Cancer Research Center. Through immunohistochemistry and recurrence status of patients after adjuvant chemotherapy, we found that PARP1 was highly expressed in the tumors of patients who relapsed after adjuvant chemotherapy (Figures 9A–C). Moreover, the recurrence time of patients with high PARP1 expression was significantly shorter than that of patients with low expression (Figures 9A–C). Thus PARP1 can be used as an important indicator to clinically predict recurrence in postoperative adjuvant chemotherapy patients, and provides confirmation that PARP1 play an important role in chemotherapy resistance.

## Discussion

We used sequence analysis of tumors from patients who relapsed after OXA chemotherapy and the patients with a good chemotherapy response. It was found that PARP1 was significantly increased in relapsed patients after postoperative chemotherapy. We then verified that PARP1 played a pivotal role in OXA resistance using OXA resistant cell lines and organoid patient derived xenografts *in vivo* BALB/C NUDE mice. In order to explore the relationship between PARP1 and OXA resistance, we inhibited PARP1 to significantly enhance the ability of OXA to kill cancer and OXA resistance cells. The combined use of PARP1 inhibitors with OXA can significantly inhibit activity of GC organoids, which affects their tumor initiation ability. *In vivo* experiments also showed that inhibiting PARP1 significantly overcame the resistance to OXA. Subsequently, we found that PARP1 mediates the DNA repair ability of OXA resistance cells by regulating the DNA repair pathway BER, and after the combination of PARP1 inhibitor, Olaparib, the joint effect allowed the drugs to effectively cause homologous recombination failure through impaired CDK1 and BRCA1 function, eventually leading to tumor cell apoptosis.

PARP1 is a multifunctional protein post – translational modifier found in most eukaryotic cells. It is activated by recognizing fragments of DNA that are structurally damaged and is thought to be a DNA damage receptor. It also performs polyadenosine diphosphate ribosylation of many nucleoproteins. Proteins modified by PARP1 include histones, RNA polymerase, DNA polymerase, and DNA ligase. ADP-ribosylation of histones results in their detachment, which is helpful to repair the binding of proteins and repair DNA damage (Alemasova and Lavrik, 2019). PARP1 plays a very important role in the BER pathway, and studies have shown that PARP1 can directly regulate the repair process during DNA single strand breakage or base resection repair (BER) (Ronson et al., 2018). The breakdown of PARP1 function inhibits the BER process (Reynolds et al., 2015). BER pathway is an important signaling pathway leading to oxaliplatin resistance (Sharma and Dianov, 2007). Our study also found that PARP1 expression could cause oxaliplatin resistance through BER by inhibiting the XRCC1. The XRCC1 also participates in MMEJ (an alternative pathway to NHEJ) but we found no significant changes in ERCC1, an important marker of MMEJ (Sfeir and Symington, 2015; Seol et al., 2018).

PARP1 inhibitors can enhance the efficacy of radiotherapy, alkylating agents and platinum-based chemotherapy by inhibiting DNA damage repair and promoting apoptosis of tumor cells (Wang et al., 2017). It wasn’t until 2014 that olaparib, the world’s first PARP (polyADP ribosome polymerase) inhibitor, was approved for the treatment of ovarian cancer, followed by the Pani family of Rucaparib, niraparib and tarazoparib (Wang et al., 2019). PARP and BRCA are both regulators of DNA repair, and PARP is responsible for single-strand repair. When PARP is inhibited, the single-strand breaks of cells continue to increase and gradually develop into double-strand breaks. At this time, BRCA is required for high-Fi precision repair (homologous recombination) of double-strand breaks to prevent cell death caused by DNA instability (D’Andrea, 2018). Although most studies have shown that PARP1 inhibitors can effectively enhance the efficacy of chemotherapy drugs, the mutation rate of BRCA1 mutations in most gastrointestinal tumors is actually not high (Narod and Foulkes, 2004).

Our study found that PARP1 inhibitors in combination with OXA have a powerful anti-tumor effect in gastric cancer patients without BRCA1 mutations. We found that CDK1 was an important factor affecting the function of PARP1 when we searched for the core gene influencing PARP1 function by means of WGCNA. Cell cycle progression is controlled by cyclin-dependent kinases (CDKs) and is a tightly regulated process in eukaryotic cells. Genomic integrity is maintained through the precise activation of CDKs and the correct timing coordination of DNA synthesis. CDK2 and CDK1 co-mediate the S and G2 phases, while CDK1 regulates the G2/M phase and mitotic progression. The deletion of CDKs in a single shRNA-mediated transformed cell suggests that they can easily complement each other (Cai et al., 2006). Exposure to genotoxic damage leads to activation of a checkpoint cascade that downregulates CDK activity and imposes cell cycle arrest to prevent the reproduction of damaged DNA. Delayed cell cycle progression is caused by DNA-induced activation of phosphatidylinositol 3 kinase-like protein kinase ATM (ataxia – telangiectasia mutation) and ATR (ATM and RAD3 associated) (Abraham, 2001). BRCA1 is an important component of ATM- and ATR-mediated checkpoint signaling and is hyperphosphorylated by ATM and ATR during DNA damage. BRCA1 acts as a scaffold to promote ATM/ATR phosphorylation of a set of substrates including CHK1 and CHK2 (Foray et al., 2003). Although CDK2 and CDK1 can compensate for each other during cell cycle progression, allowing a single CDK-depleted cell to proliferate, it is unclear whether they play a non-overlapping role in DNA damage-induced checkpoint control. In this study, we found that in the response of cisplatin to gastric cancer cells, selective inhibition of CDK1 could affect the function of BRCA1, while OXA could play an independent role, indicating that OXA could inhibit CDK1 and thus exert the function of inhibiting BRCA1. This is the first time that oxaliplatin has been found to play a role in gastric cancer by inhibiting CDK1 phosphorylation.

## Conclusion

Our study found that PARP1 inhibitors in combination with oxaliplatin have a powerful anti-tumor effect in gastric cancer patients without BRCA1 mutations. However, our study found that oxaliplatin itself can affect BRCA1 by inhibiting the function of CDK1, causing BRCA1 dysfunction and allowing PARP1 inhibitors to function effectively.

## Materials and Methods

### Cell Culture

GC cell lines AGS (ATCC^®^ CRL-1739^TM^) and MKN74 (ABC-TC0689) were ordered from the Francis Crick Institute Cell Services, SNU719 cells were provided by Nanjing Kegen Biotechnology Co., Ltd. All GC cell lines were grown in complete medium containing 10% FCS and RPMI.

In order to cultivate a stable OXA-resistant GC cell line, AGS, SNU719 and MKN74 cells were exposed to RPMI with an initial OXA (No. S1224, Selleckchem) concentration of 1 μmol.L^–1^ and 10% fetal bovine serum. The surviving cell population was grown to a concentration of 80% and passaged twice within 9 days to ensure survival. The above process was repeated for the surviving cells with consecutively higher OXA concentrations of 10 μmol.L^–1^ (15 days), 20 μmol.L^–1^ (30 days), 50 μmol.L^–1^ (60 days), 100 μmol.L^–1^ (90 days), and finally 200 μmol.L^–1^ (120 days). Afterward, resistance to OXA was confirmed by IC50 and a colony forming test (see Supplementary Figures 1A–C).

### Human Tissue and Organoids

Human GC tissues were taken from patients who underwent gastric cancer surgery in The First Affiliated Hospital of Sun Yat-sen University, PRC. They agreed and signed a donation and research consent form. This was approved by the Clinical Research and Animal Experiment Ethics Committee of The First Affiliated Hospital of Sun Yat-sen University [Ethical Review (2017) No. 208]. This research complied with all the ethics of human participation in research.

Biopsies were obtained from the surgery of gastric cancer patients treated by the Gastric Cancer Research Center of Sun Yat-sen University, for GC organoid culture. According to the patient’s postoperative clinical history, we included two cases of recurrence of GC after chemotherapy (named rGC1 and rGC2 repectively) and two cases of satisfactory post-chemotherapy outcomes for our organoid cultures (named sGC1 and sGC2). The organoids were screened through the Scientific Research Center of the Seventh Affiliated Hospital of Sun Yat-sen University and organoid strains of 4 patients were finally selected to be included in the experiment.

The organoids were generated as follows, the GC sample was placed in 50 U.mL^–1^ penicillin-streptomycin (Thermo Fisher Scientific) ice-cold G solution, was minced on ice and incubated in DMEM containing 1 mg.ml^–1^ collagenase V (Sigma-Aldrich) for 1 h at 37°C. Ice-cold PBS was added to stop the digestion, and the mixture was then centrifuged at 4°C (300 G, 5 min). The samples were further digested with TrypLE (Thermo Fisher Scientific) at 37°C for 5 min, which was then stopped with a large quantity of PBS. The suspension was filtered through 40 μm nylon mesh, centrifuged, and the cells were resuspended in the medium. Organoids were passaged with TrypLE every 2 weeks. The medium for establishing and culturing human GC organoids was as described in the literature (Seidlitz et al., 2019).

### Lentivirus Production and Infection of Organoids

Control and shRNA_PARP1-expressing pLKO vectors were purchased from Sigma (China). PARP1 overexpression vectors designed to generate the lentivirus were obtained from Shanghai Genechem Co., Ltd. All lentiviral particles were produced in HEK293T cells by standard procedures, concentrated by ultracentrifugation at 100,000*g* for 2 h and resuspended in sterile PBS. Organoids were extracted from Matrigel using TrypLE Express (Thermo Fisher Scientific), resuspended in OptiMEM with 10 μg.mL^–1^Polybrene, and then mixed with the virus solution in an incubator for 6 h. Cells were plated back into Matrigel and split 72 to 168 h later when antibiotic selection was started.

### Quantitative Real-Time PCR

When the number of cells were less than 10^3^ we used MagMAX-96 Total RNA Isolation Kit (Ambion) to extract RNA, for higher cell numbers, RNeasy Mini Kit (Qiagen) was used. Random hexamer primers (Invitrogen) were used with the SuperScript III First-Strand cDNA synthesis kit or iScript cDNA synthesis kit (BioRad) according to the manufacturer’s instructions to generate cDNA. cDNA was diluted with distilled water to 2 mol/L and RT-qPCR was performed using the Express SYBR GreenER (Thermo Fisher Scientific) ABI7500 (Applied Biosystems). The primers were designed using the Universal Probabilistic Analysis and Design Center (Roche) to ensure that they span the exon-exon junction. Actin was used for normalization. The list of RT-qPCR primers is provided in Supplementary Table 2.

### Western Blotting

The total protein of the extracted cells was lysed in ice-cold cell lysis buffer (NEB) containing 1 mM PMSF and 1:100 protease inhibitor cocktail (Sigma). The lysate was pre-cleared with 15 × l protein A Sepharose 4B beads (Sigma) at 4°C for 30 min. NE-PER^TM^ Nuclear and Cytoplasmic Extraction Reagents (Thermo Fisher Scientific, 78833) were used to extract nucleoprotein from cells. The BCA protein assy (Pierce, Rockford, IL, United States) and Western Blot procedures were performed as described previously (Ruiz et al., 2019). Antibodies used included Anti-beta Actin antibody (1:50000, Abcam, ab49900), Anti-gamma H2A.X (phospho S139) antibody (1:1000, Abcam, ab2893), PARP-1 antibody (F-2) (1: 500, Santa Cruz, sc-8007), Cdc2 p34 antibody (17) (1:500, Santa Cruz, sc-54), BRCA1 antibody (D-9) (1:500, Santa Cruz, sc-6954), Phospho-cdc2 (Tyr15) Antibody (1:1000, Cellsignal, #9111), Phospho-BRCA1 (Ser1497) Polyclonal Antibody (1:1000, Thermo Fisher Scientific, # PA5-64621), Rad51 Antibody (G-5) (1:500, Santa Cruz, sc-133089), XRCC1 (1:1000, Abcam, ab44830), and Lamin B1 (1: 20000, Proteintech, 66095-1-Ig).

### Flow Cytometry

Annexin V-PI apoptosis assay was performed using Annexin V-FITC Apoptosis Detection Kit (Sigma-Aldrich), following the protocol provided by the manufacturers. FlowJo 10 software was used to analyze the data.

### Colony Formation Assay and Cell Viability

Control (DMSO), Olaparib (No.S1060, Selleckchem) (25 μM.mL-1), OXA (10 uM.mL-1), cis Platinum (5 μM.mL-1) and CDK1 inhibitor (AG-024322, BIOQUOTR, and 837364-57-5) (0.12 μM.mL-1) were added to cells (500/well) in a 6-well plate. After 2 weeks of culturing, the formation of colonies or colosphere was evidently visible, the cell colonies were then fixed, stained with 0.1% crystal violet in 20% methanol solution, and counted. This process was repeated three times per solution type.

In a 96-well transparent bottom blackboard, 3,000 cells were planted in each well (organoids were planted in Matrigel). The drug was then added to each well according to a 10-fold concentration gradient. Cell viability as determined by Adenosine triphosphate (ATP) levels (Promega, Madison, WI, United States) were assayed by CellTiter-Glo using a luminometer (PerkinElmer Life and Analytical Sciences, Boston, MA, United States) 48 h later.

### Immunohistochemical Staining

The tissues were collected, fixed with 10% neutral buffered formalin (NBF, Sigma) for 16 h, dehydrated with 70% ethanol, and embedded in 4 × m paraffin sections. H&E staining was performed according to standard procedures. After heat-mediated antigen extraction in 10 mM sodium citrate buffer (pH 6.2), the endogenous peroxidase was blocked with 1.6% hydrogen peroxide, and PARP-1 (Proteintech, 13371-1 -AP), KI67 (Abcam, ab15580), Caspase 3 (Proteintech, 19677-1-AP), BRCA1 (Affinity Biosciences, AF6289), Phospho-BRCA1-Ser1497 (Affinity Biosciences, AF8204), CDK1 (Abcam, ab133327), and Phospho-CDK1-Y15 (Abclonal, AP0016) were stained with DAB according to manufacturer’s manual. Positive cells were counted in 5 random fields of view per slide.

### Immunofluorescent Staining

Cell/organoids were grown in a glass bottom tissue culture plate (Ibidi, lot:191218/2), fixed with 5% NBF for 10 min, and blocked with PBS containing 10% FCS, 1% BSA (Sigma) and 0.2% Triton-X. The primary antibody was incubated in blocking buffer at 4°C for 16 h. The fluorescent secondary antibody was incubated with 3 μM DAPI in blocking buffer at 20°C for 1–6 h. Fluorescence staining was imaged on a Zeiss LSM 780 confocal microscope. Tissues were prepared as detailed for Immunohistochemical staining. Secondary antibodies were fluorophore-conjugated and incubated with 3μM DAPI in the dark. Before mounting, slides were incubated in 0.1% (w/v) Sudan black B (Sigma) in 70% ethanol to reduce background signal. Antibodies include Anti-gamma H2A.X (phospho S139) antibody (1:1000, Abcam, ab2893), PARP-1 antibody (F-2) (1: 500, Santa Cruz, sc-8007), Phospho-cdc2 (Tyr15) Antibody (1:1000, Cellsignal, #9111), Phospho-BRCA1 (Ser1497) Polyclonal Antibody (1:1000, Thermo Fisher Scientific, # PA5-64621), Rad51 Antibody (G-5) (1:500, Santa Cruz, sc-133089), XRCC1 (1:1000, Abcam, ab44830), and XRCC1 (1:1000, Abcam, ab235196).

### The PDOX Mouse Model

*In vivo* experiments were performed in accordance with the Institutional Animal Care and Use Committee (IACUC) regulations. The experimental protocol was approved by the Clinical Research and Animal Experiment Ethics Committee of The First Affiliated Hospital of Sun Yat-sen University [Ethical Review (2017) No. 208]. The experiment was performed by the staff of the Animal Center of the First Affiliated Hospital of Sun Yat-sen University. In order to study the tumorigenesis ability of OXA resistance, 100,000 cells previously selected were inoculated into BALB/C NUDE female mice with Matrigel (BD, 354230). After 25 days, 6 mice with organoids transplantation tumors received a treatment of OXA (Selleckchem, s1224) at a dose of 5 mg.kg^–1^ twice a week for the period of 4 weeks.

For the other 6 mice, PBS were injected intraperitoneally. The cancer-bearing BALB/C NUDE mice were sacrificed 4 weeks later, and tumors were harvested for measuring and weighing. In order to study the drug resistance of PARP1 expression, 100,000 cells of plko and PARP1-sh1 (rGC1 and rGC2) were inoculated into BALB/C NUDE mice with Matrigel (BD, 354230). After 25 days, 6 mice with organoids transplantation tumors received OXA treatment as before. The cancer-bearing BALB/C NUDE mice were sacrificed 4 weeks later, and tumors were harvested for measuring and weighing. we inoculated 200,000 cells of control and PARP1 overexpression (sGC1 and sGC2) into BALB/C NUDE mice with Matrigel (BD, 354230). After 25 days, 6 mice with organoids transplantation tumors received a treatment of OXA (Selleckchem, s1224) at a dose of mg.kg^–1^ twice a week for the period of 4 weeks. The cancer-bearing BALB/C NUDE mice were sacrificed 4 weeks later, and tumors were harvested for measuring and weighing.

The organoids of rGC1 and rGC2 were digested into single cells by TrypLE and then counted, and 100,000 cells were mixed with Matrigel and inoculated subcutaneously into BALB/C NUDE mice (6 per group). After 25 days, the organoids transplanted BALB/C NUDE mice received intraperitoneal injection of either OXA (Selleckchem, s1224) + Olaparib (Selleckchem, AZD2281, s1060), OXA, Olaparib, or PBS. OXA dose was 5 mg.kg^–1^, Olaparib dose was 50 mg.kg^–1^, combined group dose was mg.kg^–1^ of OXA and 25 mg.kg^–1^ of Olaparib twice per week, each treatment lasting for the period of 4 weeks. The tumor size and body mass of the mice were measured every 3 days. The mice were sacrificed 1 month later, and tumors were removed. All tumors were photographed and the mass and volume determined. Tumor volume (mm^3^) = 0.5 × width^2^ × length.

### RNA Isolation and Microarray

Total RNA was extracted from tissue samples, and Nanodrop 2000 was used to detect the concentration and purity of the RNA. Agarose gel electrophoresis was used to detect RNA integrity, and Agilent 2100 was used to determine the RIN value. A single library construction required that the total amount of RNA was no less than 5 μg, the concentration ≥ 200 ng.μL^–1^, and the OD260/280 between 1.8 and 2.2. The mRNA capture and library preparation were completed by the advanced sequencing equipment of Shanghai Origin-gene Biomedical Technology Co., Ltd. using KAPA mRNA HyperPrep kit (Roche). The biological triplicate libraries were sequenced on the Illumina Truseq TM RNA sample prep Kit platform of the facility, and each sample produced an average of 25 million single-ended reads of 75 bp. The designated reference genome was used to align the high-quality sequence with post quality control. The PDOX sample was first compared with mouse reference genome. After removing mice-related data, it was then compared with human reference genome. The human reference genome was obtained from Ensembl database, genome version GRCh38, gene annotation information was Ensemble 92. Before alignment, cutadapt (version 1.9.1) was used for quality control and adaptor trimming of the original reading. Annotation release 86 was used to sequence the reads of the human genome GRCh38 using RSEM 1.3.0 and STAR 2.5.2, and count the subsequent gene levels. In version 3.6.1 of R package, the DESeq2 package (version 1.24.0) was used for normalization and differential expression analysis of raw count data. Regularized logarithmic transformation was performed on the rlog function.

### Clinical GC Patient Samples

From May 2010 to February 2020, the progressive GC tissue samples before the start of OXA treatment were collected from the First Affiliated Hospital of Sun Yat-sen University (*n* = 100) through surgical specimens or biopsy, and the patients’ research consent form were signed and documented. This study was approved by the Ethics Committee of The First Affiliated Hospital of Sun Yat-sen University [Ethical Review (2018) No. 087]. The OXA group received at least 6 cycles of OXA treatment. The detailed clinical characteristics of the patients can be found in Table 1. The tumor response to chemotherapy was evaluated by the three-dimensional volume reduction rate or tumor response rate (radiological evaluation), and evaluated in accordance with the response evaluation criteria in the solid tumor (RECIST) guidelines (Eisenhauer et al., 2009). In the validation phase, patients with worsening symptoms, new lesions, or radiologically assessed tumor regeneration ≥ 25% were assigned to the progressive disease (PD) group (*n* = 45) and the remaining non-PD group (*n* = 55). PFS is defined as the duration from tumor resection to PD. Follow-up was performed every 3 months (for the initial 0–2 years), 6 months (subsequent 2–4 years), and once a year until death or February 2020. The follow-up study included abdominal computed tomography and postoperative physical examination.

**TABLE 1:** Demographics of GC patients of SYSU.

	Non-progress (55)	Progress (45)	*P*
Gender			
Male	29	24	0.952
Female	26	21	
Age			
≤65	15	20	0.060
>65	40	24	
M staging			
M0	55	45	–
M1	0	0	
T staging			
T1	1	3	0.370
T2	19	2	
T3	31	31	
T4	4	9	
N staging			
N0	27	10	0.343
N1	20	15	
N2	5	9	
N3	3	11	
Differentiation			
High	31	31	0.130
Moderately	18	11	
Poorly	6	3	
Undifferentiation	0	0	
PARP1			
High	43	26	0.032
Low	12	19	
CD133			
High	39	22	0.025
Low	16	23	

### Patient Information in Public Databases

The transcriptome data of patients with gastric adenocarcinoma confirmed by pathology was downloaded from the TCGA website^1^ in June 2020, including data from 416 patients with gastric adenocarcinoma and general information of the corresponding cases. Data that did not list survival time were excluded, leaving 416 cases of gastric cancer and 33 cases of adjacent tissues. Inclusion criteria: (a) diagnosis age ≥ 8 years old; (b) tumor site: stomach; (3) cases with clear pathology. The exclusion criteria are as followed: (a) multiple tumor; (b) carcinoma *in situ*; (c) incomplete follow-up data; and (d) deaths within 30 days. Proteomics data of patients with gastric adenocarcinoma were downloaded from the CPTAC website^2^ in June 2020, including data and corresponding general information of 130 gastric adenocarcinoma patients.

### Gene Set Enrichment Analysis (GSEA)

Gene set enrichment analysis was performed using software (GSEA V4.0.3) developed by the Broad Institute of MIT and Harvard University^3^. The RNA-seq datasets of OXA resistance patients, normalized RNA read counts were used for analysis, and the following settings were applied: permutation number = 1000, permutation type = gene set, enrichment statistics = weighting, a measure of gene ranking = signal noise. For the TCGA gastric cancer dataset, the samples were grouped according to their expression above or below the median value. The normalized RSEM read count was used for analysis, and the following settings were applied: number of permutations = 1000, permutation type = phenotype, enrichment statistics = weighting, measurement of gene ranking = signal 2 noise. Recognized marker gene set 40, KEGG pathway or gene ontology (GO) terms, and false discovery rate (FDR q) < 0.05 were considered significant enrichment.

### Screening of Differentially Expressed Genes (DEGs)

The expectation-maximization method RNA-Seq was used to normalize the 3-level transcriptome data of the data set, and the logarithmic transformation of all gene expression values was performed. Approximate data were normally distributed after normalization by quantiles (Li and Dewey, 2011). In this study, the R package limma program v3.28.14 was used to analyze the differential genes of gene expression data, and its mRNA satisfied *P* < 0.01, false discovery rate (FDR) < 0.01 and | log2 fold change (FC)| > 1.5, where *P* < 0.05 indicated that the hypothesis test was statistically significant. FDR is a control indicator for the error rate of the hypothesis test. As an evaluation index of the selected differential genes, the number of false rejections was proportional to the number of rejected invalid hypotheses. FC was usually used to describe the degree of change from the initial value to the final value. In this study, the ratio of tumor tissue gene expression value to normal tissue gene expression value was used, also known as the fold change. The heatmap and volcano map of the differential genes were constructed in R language for visual comparison.

### WGCNA Co-expression Network Construction

Gene expression data (mRNA-seq data) was downloaded from the TCGA database. A total of 24,991 genes were identified in each sample. Analysis of variance was performed and then sorted from largest to smallest. The SD value of each gene was calculated and sorted from largest to smallest, and then the top 5000 genes were selected for WGCNA. WGCNA package in R software was used to construct a gene co-expression network from the expression data map of these 5000 genes (Luo et al., 2015). Using the adjacency function in WGCNA, an adjacency matrix was constructed by calculating the Pearson correlation between all pairs of genes in the selected sample. In this study, β = 7 (scale-free R2 = 0.9) was used as the soft threshold parameter to ensure a scale-free network. In order to further identify the functional modules in the co-expression network of these 5000 genes, the adjacency matrix was used to calculate the Topological Overlap Measure (TOM), which represents the overlap in the shared neighborhood. We identified related modules by calculating the correlation between MEs and PARP1 expression levels. Then the log10 transformation of the *p* value (GS = lgP) in the linear regression of gene expression and clinical PARP1 expression level information was defined as gene significance (GS). In addition, module significance (MS) is defined as the average GS of all genes in a module. In general, among all the selected modules, the module with the highest absolute value of MS was considered to be the module related to the level of PARP1 expression.

### PPI Network Construction of Key Module Gene

The Hub gene, which is highly interconnected with the nodes in the module, is considered to have important functions. We selected the top 30 Hub genes in the module network as candidate genes for further analysis and verification. The STRING data set is an online biological resource that can decode the interaction between proteins and proteins to obtain the actual precise functions of proteins (Snel et al., 2000). The candidate gene was submitted to the protein interaction of STRING, and the binding confidence interval of the cutoff value was set to 0.4. In the plugin, Molecular Complex Detection (MCODE), the significant models with strong protein-protein connection were calculated and selected with the default parameters (degree cut ≥ 2, node score cut ≥ 2, K-core ≥ 2, maximum depth = 100). *P* < 0.05 was considered statistically significant.

### Statistical Analysis

The images and graphs shown represent several experiments repeated on different individuals at different times. Each experiment was repeated independently at least three times. All statistics were performed using SPSS and R software. The statistical test was explained in the figure legend. All results were statistically different based on the mean ± SD, *P* < 0.05.

## Data Availability Statement

The datasets presented in this study can be found in online repositories. The names of the repository/repositories and accession number(s) can be found below: BioProject PRJNA669415.

## Ethics Statement

This study was approved by the Ethics Committee of The First Affiliated Hospital of Sun Yat-sen University [Ethical Review (2018) No. 087] and the Clinical Research and Animal Experiment Ethics Committee of The First Affiliated Hospital of Sun Yat-sen University [Ethical Review (2017) No. 208].

## Author Contributions

AB, YH, CZ, HL, and CW designed the study. HL, CW, and LL contributed to development of methodology. HL, CW, LL, IE, and ZZ contributed to acquisition of data (provided animals, acquired and managed patients, and provided facilities, etc.). HL, CW, LL, and ER contributed to analysis and interpretation of data (e.g., statistical analysis, biostatistics, and computational analysis). JO, RR, HL, CW, WC, ZH, and LY contributed to cultivation and development organoid. All authors contributed to writing the manuscript.

## Conflict of Interest

The authors declare that the research was conducted in the absence of any commercial or financial relationships that could be construed as a potential conflict of interest.

## Publisher’s Note

All claims expressed in this article are solely those of the authors and do not necessarily represent those of their affiliated organizations, or those of the publisher, the editors and the reviewers. Any product that may be evaluated in this article, or claim that may be made by its manufacturer, is not guaranteed or endorsed by the publisher.

## Footnotes

1
https://portal.gdc.cancer.gov/

2
https://cptac-data-portal.georgetown.edu/study-summary/S025

3
https://www.gseamsigdb.org/gsea/index.jsp

## Supplementary Material

Supplementary Figure 1 Verifying the drug resistance level in GC resistant strains. **(A–C)** indicate the comparison of cell viability between AGS, SNU719, and MNK74 Oxaliplatin resistance strains and wild-type cell strains under the effects of Oxaliplatin, respectively. **(D)** indicate the comparison of cell viability between sGC1, sGC2 and rGC1, rGC2 under the effects of Oxaliplatin, respectively. The Student’s *t* test was used for statistical analysis. Error bars indicate mean ± standard deviation. OXA, Oxaliplatin. OLP, Olaparib. CON, control group. PCDK1, CDK1 phosphorylation antibody. PBRCA1, BRCA1 phosphorylation antibody. AGSRE, AGS Oxaliplatin resistance. SNU719RE, SNU719 Oxaliplatin resistance. MKN74RE, MKN74 Oxaliplatin resistance. All experiments had repeated three times.

Supplementary Figure 2 PARP1 expression is an important mediating factor for Oxaliplatin resistance. **(A,B)** The effects of Olaparib + Oxaliplatin and Oxaliplatin on colony formation of overexpressed and normally expressed PARP1 in SNU719, MKN74, and AGS stable strains. Colonies are stained with crystal violet. **(C,D)** After treatment with Olaparib + Oxaliplatin, the BALB/C NUDE mice expression was enriched in oxidative phosphorylation, PPAR Signaling pathway. **(E–H)** After treatment with Oxaliplatin, the BALB/C NUDE mice expression was enriched in JAK STAT signaling pathway, MAPK signaling pathway, NOTCH signaling pathway, and WNT Signaling pathway. Student’s *t* test was used for statistical analysis. Error bars indicate mean ± standard deviation. AGSRE, AGS Oxaliplatin resistance. SNU719RE, SNU719 Oxaliplatin resistance. MKN74RE, MKN74 Oxaliplatin resistance. ***< 0.001. All experiments had repeated three times.

Supplementary Figure 3 PARP1 was significantly expression than XRCC1 in oxaliplatin resistant GC. **(A)** Verification by WB on the PARP1 knock-down and overexpression. **(B)** Volcano plot for the statistical significance (*Y*-axis, −log2 transformation) versus the fold change of gene expression (*X*-axis, log2 transformation) between sGC1 and sGC2, and rGC1 and rGC2 populations determined by RNA-seq (*n* = 3). FC, fold change; padj, false-discovery-rate adjusted *p* value. **(C)** RT-qPCR was used to compare PARP1 mRNA expression in SNU719, MKN74, and AGS resistant strains and wild-type cell lines. Student’s *t* test was used for statistical analysis. Error bars indicate mean ± standard deviation. OXA, Oxaliplatin. OLP, Olaparib. CON, control group. AGSRE, AGS Oxaliplatin resistance. SNU719RE, SNU719 Oxaliplatin resistance. MKN74RE, MKN74 Oxaliplatin resistance. ns, No significant. All experiments had repeated three times.

Supplementary Figure 4 CDK1 is an important target of Oxaliplatin combined with PARP1 inhibitor that can effectively kill Oxaliplatin resistance cells. **(A)** The clustering was based on the expression data in TCGA. The top 5000 genes with the highest SD values were used for the analysis by WGCNA. The color intensity was proportional to expression status (PARP1 low and PARP1 high). **(B)** Analysis of the scale-free fit index for various soft-thresholding powers (β). Analysis of the mean connectivity for various soft-thresholding powers. In all, 3 was the most fit power value. **(C)** The cluster dendrogram of module eigengenes. **(D)** The cluster dendrogram of genes in TCGA. Each branch in the figure represents one gene, and every color below represents one co-expression module. **(E)** Heatmap of the correlation between module eigengenes and the expression status of PARP1. The dark orange module was the most positively correlated with PARP1 high expression. **(F)** Hierarchical clustering of module hub genes that summarizes the modules yielded in the clustering analysis. **(G)** Heatmap plot of the adjacencies in the hub gene network. Scatter plot of module eigengenes in the Brown module. **(H)** STING database protein interaction network diagram of Brown module. Edges represent protein-protein associations. Cambridge blu, curated databases; Violet, experimentally determined; Green, gene neighborhood; Red, gene fusions; Blue, gene co-occurrence; Reseda, text mining; Black, co-expression; Lilac, protein homology. **(J)** statistical ranking of **(I)** in accordance to the number of interactive network nodes. The abscissa represents the number of nodes. All experiments had repeated three times.

Supplementary Figure 5 Treatment of Oxaliplatin inhibits HR repair pathways via blocking CDK1-BRCA1 activities in Oxaliplatin resistance gastric cancer **(A)** representative images of immunofluorescent phosporylation staining comparisons of Olaparib + Oxaliplatin, Oxaliplatin, Olaparib, and blank control group and immunofluorescent phosphorylation staining of RAD51, XRCC1 and γH2AX of MKN74, SNU719, AGS resistant strains. The scale represents 2 um. **(B)** Statistical analysis of RAD51 + cells in A. **(C)** Statistical analysis of γH2AX + cells in **(A)**. **(D)** Statistical analysis of XRCC1 + cells in A. The Student’s *t* test was used for statistical analysis. Error bars indicate mean ± standard deviation. OXA, Oxaliplatin; OLP, Olaparib; CON, control group; AGSRE, AGS Oxaliplatin resistance; SNU719RE, SNU719 Oxaliplatin resistance; MKN74RE, MKN74 Oxaliplatin resistance. ^∗∗^< 0.01, and ^∗∗∗^< 0.001. All experiments had repeated three times.
