# Correction: PARP1 inhibitor combined with oxaliplatin efficiently suppresses oxaliplatin resistance in gastric cancer-derived organoids via homologous recombination and the base excision repair pathway

**DOI:** 10.3389/fcell.2025.1672461

**Published:** 2025-08-28

**Authors:** Huafu Li, Chunming Wang, Linxiang Lan, Wenhui Wu, Ian Evans, E. Josue Ruiz, Leping Yan, Zhijun Zhou, Joaquim M. Oliveira, Rui L. Reis, Zhenran Hu, Wei Chen, Axel Behrens, Yulong He, Changhua Zhang

**Affiliations:** ^1^ Digestive Diseases Center, The Seventh Affiliated Hospital of Sun Yat-sen University, Shenzhen, China; ^2^ Adult Stem Cell Laboratory, The Francis Crick Institute, London, United Kingdom; ^3^ The Institute of Cancer Research, London, United Kingdom; ^4^ Department of Gastrointestinal Surgery, The First Affiliated Hospital of Sun Yat-sen University, Guangzhou, China; ^5^ Center of Scientific Research, The Seventh Affiliated Hospital of Sun Yat-sen University, Shenzhen, China; ^6^ Department of Medicine, The University of Oklahoma Health Sciences Center, Oklahoma City, OK, United States; ^7^ 3B’s Research Group, I3Bs – Research Institute on Biomaterials, Biodegradables and Biomimetics, Headquarters of the European Institute of Excellence on Tissue Engineering and Regenerative Medicine, AvePark, Parque de Ciência e Tecnologia, Zona Industrial da Gandra, University of Minho, Guimarães, Portugal; ^8^ ICVS/3B’s – PT Government Associate Laboratory, Guimarães, Portugal

**Keywords:** gastric cancer, L-OHP resistance, homologous recombination, PARP1 inhibitors, organoid

There was a mistake in Figures 2, 4 as published. The wrong images were erroneously used for Figures 2G, 4A. The corrected Figures 2, 4 appear below.

**FIGURE 2:**
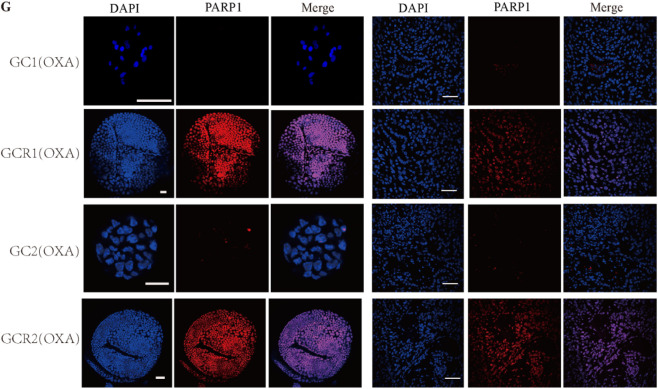
PARP1 is upregulated in oxaliplatin resistance gastric cancer. **(G)** Representative images of PARP1 levels stained by immunofluorescence in organoid and tumors. The scale bar represents 20 µm for tumour images, 200 µm for organoid images.

**FIGURE 4:**
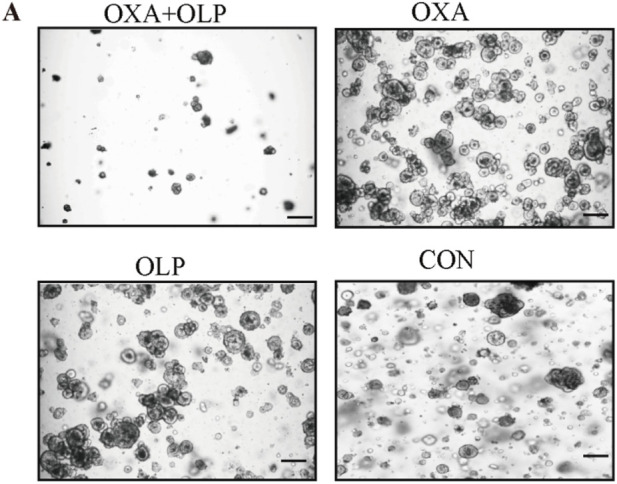
PARP1 inhibition by Olaparib sensitizes gastric cancer to Oxaliplatin. (A–C) rGC1 organoids were treated with Olaparib + Oxaliplatin, Oxaliplatin, and Olaparib, respectively, before imaging **(A)**, number of organoids (B), and size of organoids (C). The scale represents 500 µm.

The original version of this article has been updated.

