# Supplementary figures and images for: PARP1 Inhibitor Combined With Oxaliplatin Efficiently Suppresses Oxaliplatin Resistance in Gastric Cancer-Derived Organoids via Homologous Recombination and the Base Excision Repair Pathway
Source: Front Cell Dev Biol. 2021 Aug 23;9:719192. doi: 10.3389/fcell.2021.719192 (PMC8419238; doi:10.3389/fcell.2021.719192)

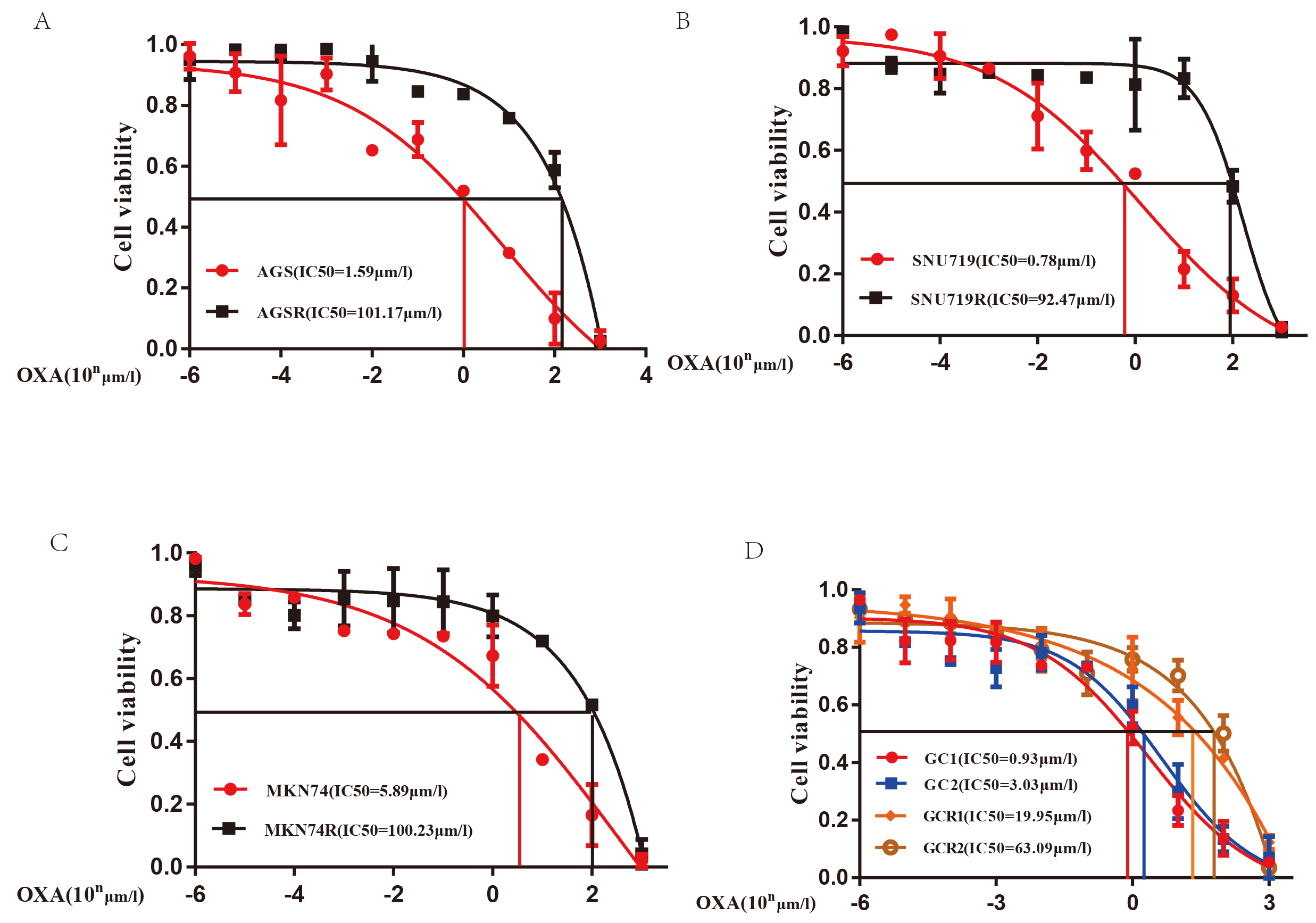

Supplement: Supplementary Figure 1 — Verifying the drug resistance level in GC resistant strains. (A–C) indicate the comparison of cell viability between AGS, SNU719, and MNK74 Oxaliplatin resistance strains and wild-type cell strains under the effects of Oxaliplatin, respectively. (D) indicate the comparison of cell viability between sGC1, sGC2 and rGC1, rGC2 under the effects of Oxaliplatin, respectively. The Student’s t test was used for statistical analysis. Error bars indicate mean ± standard deviation. OXA, Oxaliplatin. OLP, Olaparib. CON, control group. PCDK1, CDK1 phosphorylation antibody. PBRCA1, BRCA1 phosphorylation antibody. AGSRE, AGS Oxaliplatin resistance. SNU719RE, SNU719 Oxaliplatin resistance. MKN74RE, MKN74 Oxaliplatin resistance. All experiments had repeated three times. [file Image_1.tif]

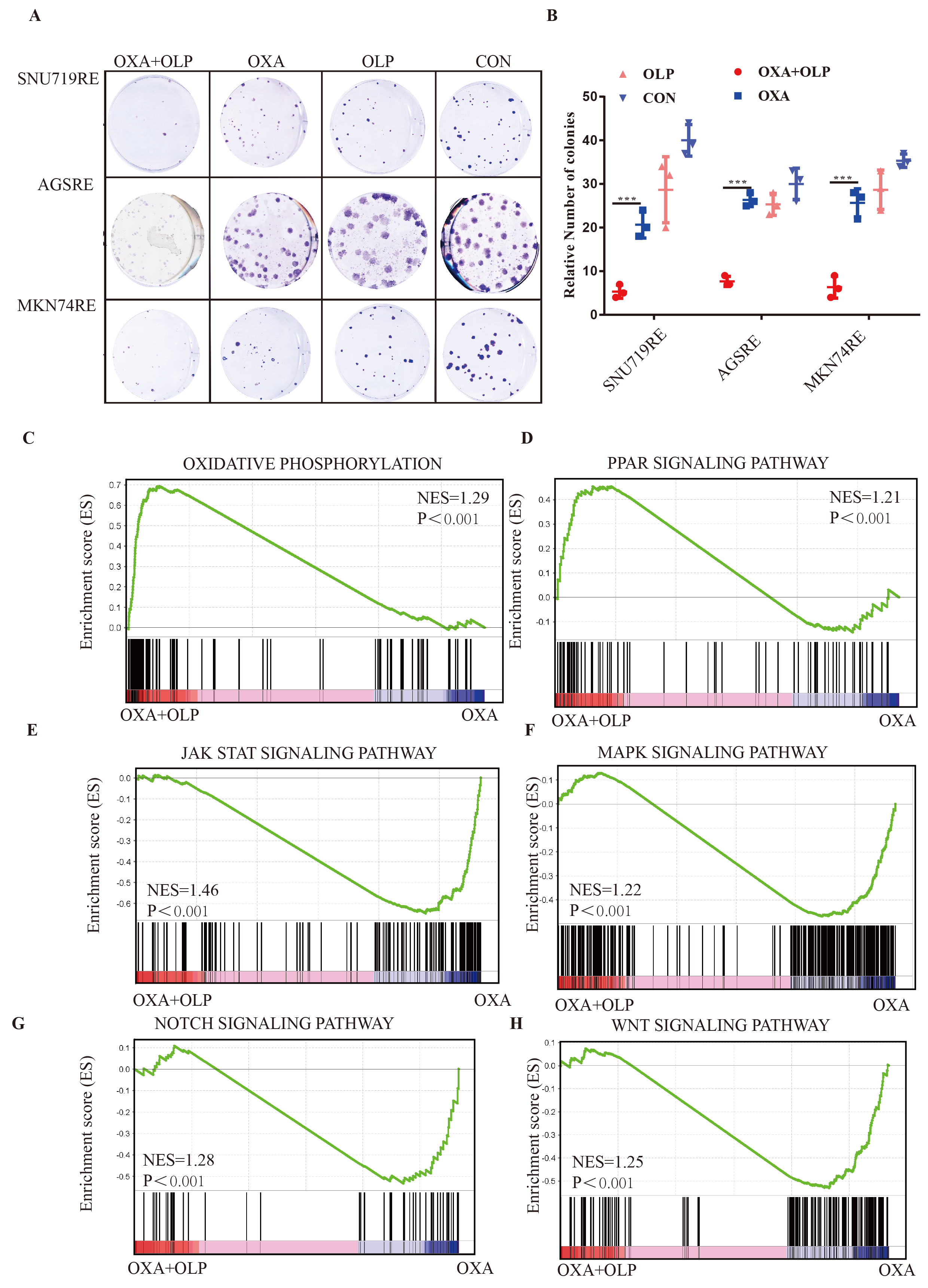

Supplement: Supplementary Figure 2 — PARP1 expression is an important mediating factor for Oxaliplatin resistance. (A,B) The effects of Olaparib + Oxaliplatin and Oxaliplatin on colony formation of overexpressed and normally expressed PARP1 in SNU719, MKN74, and AGS stable strains. Colonies are stained with crystal violet. (C,D) After treatment with Olaparib + Oxaliplatin, the BALB/C NUDE mice expression was enriched in oxidative phosphorylation, PPAR Signaling pathway. (E–H) After treatment with Oxaliplatin, the BALB/C NUDE mice expression was enriched in JAK STAT signaling pathway, MAPK signaling pathway, NOTCH signaling pathway, and WNT Signaling pathway. Student’s t test was used for statistical analysis. Error bars indicate mean ± standard deviation. AGSRE, AGS Oxaliplatin resistance. SNU719RE, SNU719 Oxaliplatin resistance. MKN74RE, MKN74 Oxaliplatin resistance. ***< 0.001. All experiments had repeated three times. [file Image_2.tif]

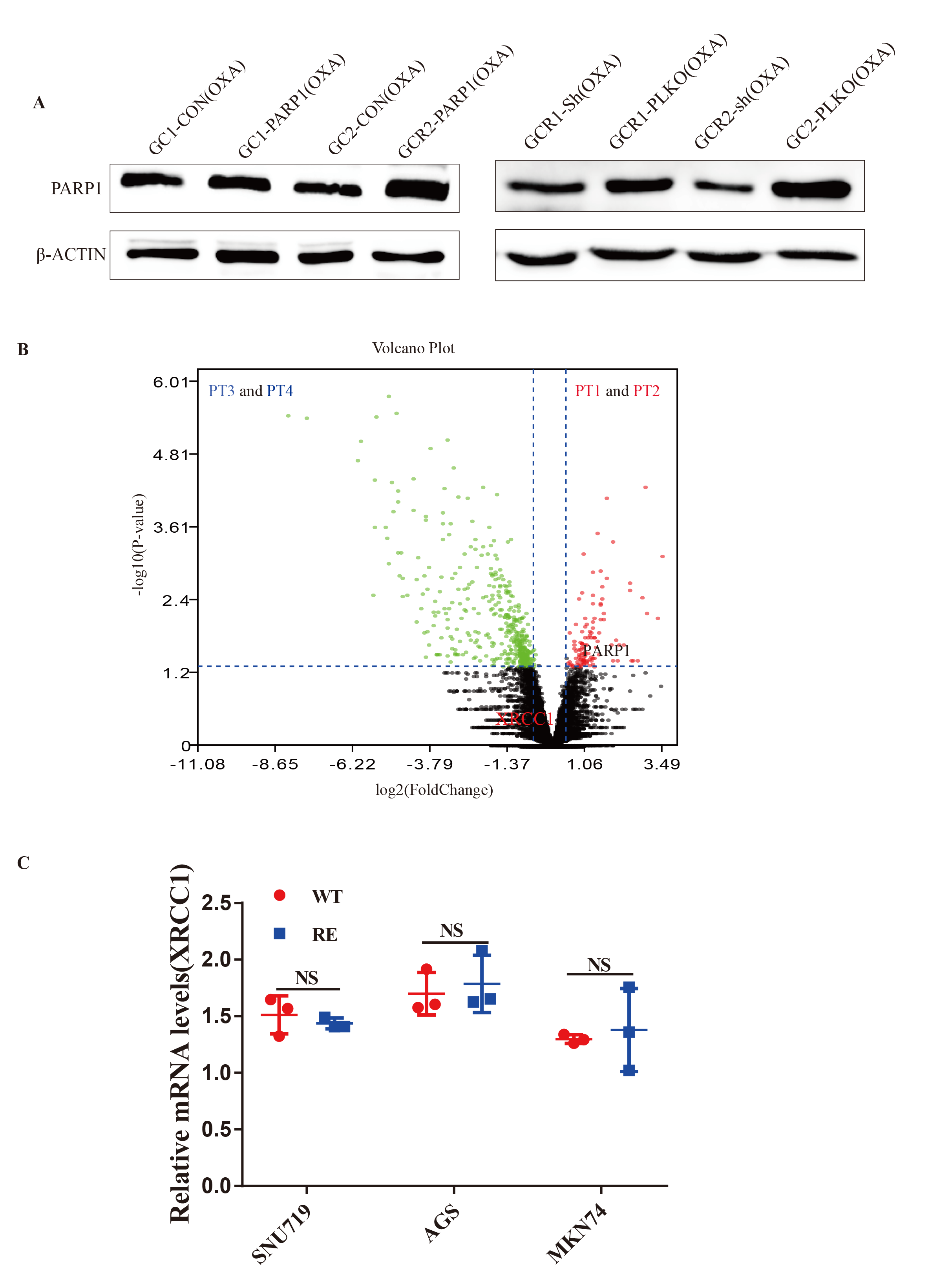

Supplement: Supplementary Figure 3 — PARP1 was significantly expression than XRCC1 in oxaliplatin resistant GC. (A) Verification by WB on the PARP1 knock-down and overexpression. (B) Volcano plot for the statistical significance (Y-axis, −log2 transformation) versus the fold change of gene expression (X-axis, log2 transformation) between sGC1 and sGC2, and rGC1 and rGC2 populations determined by RNA-seq (n = 3). FC, fold change; padj, false-discovery-rate adjusted p value. (C) RT-qPCR was used to compare PARP1 mRNA expression in SNU719, MKN74, and AGS resistant strains and wild-type cell lines. Student’s t test was used for statistical analysis. Error bars indicate mean ± standard deviation. OXA, Oxaliplatin. OLP, Olaparib. CON, control group. AGSRE, AGS Oxaliplatin resistance. SNU719RE, SNU719 Oxaliplatin resistance. MKN74RE, MKN74 Oxaliplatin resistance. ns, No significant. All experiments had repeated three times. [file Image_3.tif]

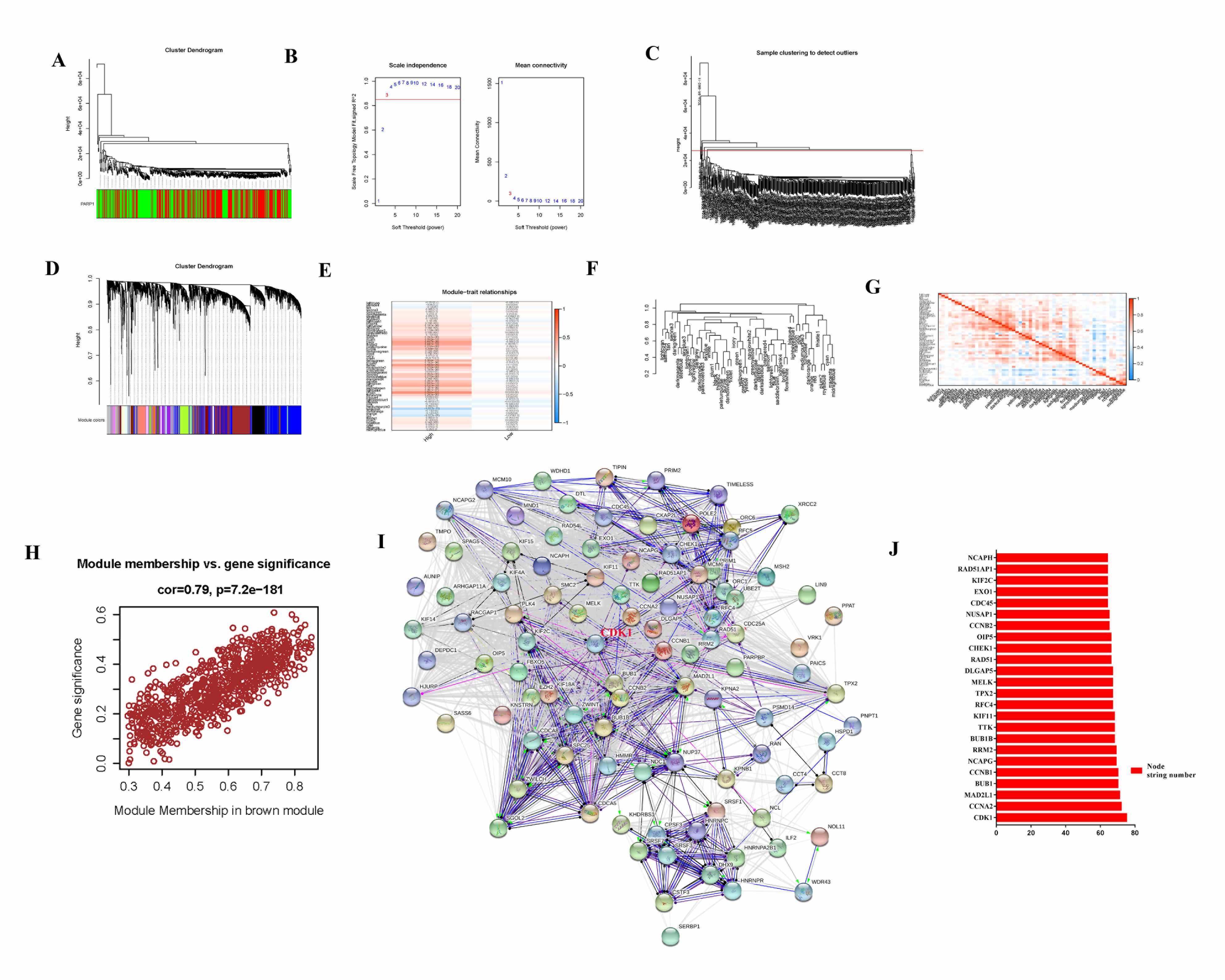

Supplement: Supplementary Figure 4 — CDK1 is an important target of Oxaliplatin combined with PARP1 inhibitor that can effectively kill Oxaliplatin resistance cells. (A) The clustering was based on the expression data in TCGA. The top 5000 genes with the highest SD values were used for the analysis by WGCNA. The color intensity was proportional to expression status (PARP1 low and PARP1 high). (B) Analysis of the scale-free fit index for various soft-thresholding powers (β). Analysis of the mean connectivity for various soft-thresholding powers. In all, 3 was the most fit power value. (C) The cluster dendrogram of module eigengenes. (D) The cluster dendrogram of genes in TCGA. Each branch in the figure represents one gene, and every color below represents one co-expression module. (E) Heatmap of the correlation between module eigengenes and the expression status of PARP1. The dark orange module was the most positively correlated with PARP1 high expression. (F) Hierarchical clustering of module hub genes that summarizes the modules yielded in the clustering analysis. (G) Heatmap plot of the adjacencies in the hub gene network. Scatter plot of module eigengenes in the Brown module. (H) STING database protein interaction network diagram of Brown module. Edges represent protein-protein associations. Cambridge blu, curated databases; Violet, experimentally determined; Green, gene neighborhood; Red, gene fusions; Blue, gene co-occurrence; Reseda, text mining; Black, co-expression; Lilac, protein homology. (J) statistical ranking of (I) in accordance to the number of interactive network nodes. The abscissa represents the number of nodes. All experiments had repeated three times. [file Image_4.tif]

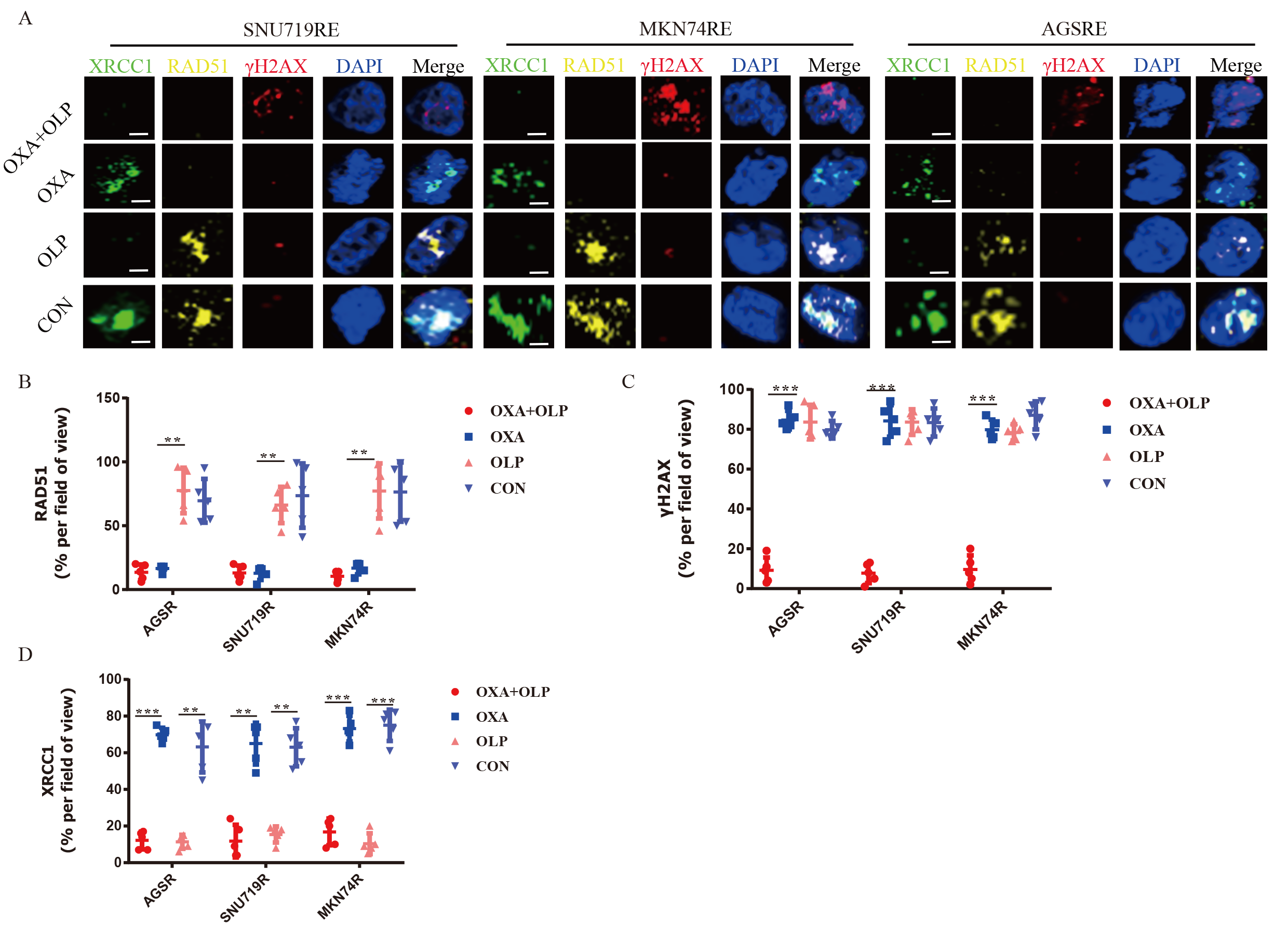

Supplement: Supplementary Figure 5 — Treatment of Oxaliplatin inhibits HR repair pathways via blocking CDK1-BRCA1 activities in Oxaliplatin resistance gastric cancer (A) representative images of immunofluorescent phosporylation staining comparisons of Olaparib + Oxaliplatin, Oxaliplatin, Olaparib, and blank control group and immunofluorescent phosphorylation staining of RAD51, XRCC1 and γH2AX of MKN74, SNU719, AGS resistant strains. The scale represents 2 um. (B) Statistical analysis of RAD51 + cells in A. (C) Statistical analysis of γH2AX + cells in (A). (D) Statistical analysis of XRCC1 + cells in A. The Student’s t test was used for statistical analysis. Error bars indicate mean ± standard deviation. OXA, Oxaliplatin; OLP, Olaparib; CON, control group; AGSRE, AGS Oxaliplatin resistance; SNU719RE, SNU719 Oxaliplatin resistance; MKN74RE, MKN74 Oxaliplatin resistance. ∗∗< 0.01, and ∗∗∗< 0.001. All experiments had repeated three times. [file Image_5.tif]
